# Synaptic alterations associated with disrupted sensory encoding in a mouse model of tauopathy

**DOI:** 10.1093/braincomms/fcae134

**Published:** 2024-04-15

**Authors:** Soraya Meftah, Annalisa Cavallini, Tracey K Murray, Lukasz Jankowski, Suchira Bose, Michael C Ashby, Jonathan T Brown, Jonathan Witton

**Affiliations:** Faculty of Health and Life Sciences, Department of Clinical and Biomedical Science, University of Exeter, Exeter, EX1 2LU, UK; School of Physiology, Pharmacology & Neuroscience, University of Bristol, Bristol, BS8 1TD, UK; Erl Wood Manor, Eli Lilly Pharmaceuticals, Windlesham, Surrey, GU20 6PH, UK; Erl Wood Manor, Eli Lilly Pharmaceuticals, Windlesham, Surrey, GU20 6PH, UK; Erl Wood Manor, Eli Lilly Pharmaceuticals, Windlesham, Surrey, GU20 6PH, UK; Erl Wood Manor, Eli Lilly Pharmaceuticals, Windlesham, Surrey, GU20 6PH, UK; School of Physiology, Pharmacology & Neuroscience, University of Bristol, Bristol, BS8 1TD, UK; Faculty of Health and Life Sciences, Department of Clinical and Biomedical Science, University of Exeter, Exeter, EX1 2LU, UK; Faculty of Health and Life Sciences, Department of Clinical and Biomedical Science, University of Exeter, Exeter, EX1 2LU, UK; School of Physiology, Pharmacology & Neuroscience, University of Bristol, Bristol, BS8 1TD, UK

**Keywords:** synapses, glutamatergic, tauopathy, somatosensory, neurodegeneration

## Abstract

Synapse loss is currently the best biological correlate of cognitive decline in Alzheimer’s disease and other tauopathies. Synapses seem to be highly vulnerable to tau-mediated disruption in neurodegenerative tauopathies. However, it is unclear how and when this leads to alterations in function related to the progression of tauopathy and neurodegeneration. We used the well-characterized rTg4510 mouse model of tauopathy at 5–6 months and 7–8 months of age, respectively, to study the functional impact of cortical synapse loss. The earlier age was used as a model of prodromal tauopathy, with the later age corresponding to more advanced tau pathology and presumed progression of neurodegeneration. Analysis of synaptic protein expression in the somatosensory cortex showed significant reductions in synaptic proteins and NMDA and AMPA receptor subunit expression in rTg4510 mice. Surprisingly, *in vitro* whole-cell patch clamp electrophysiology from putative pyramidal neurons in layer 2/3 of the somatosensory cortex suggested no functional alterations in layer 4 to layer 2/3 synaptic transmission at 5–6 months. From these same neurons, however, there were alterations in dendritic structure, with increased branching proximal to the soma in rTg4510 neurons. Therefore, *in vivo* whole-cell patch clamp recordings were utilized to investigate synaptic function and integration in putative pyramidal neurons in layer 2/3 of the somatosensory cortex. These recordings revealed a significant increase in the peak response to synaptically driven sensory stimulation-evoked activity and a loss of temporal fidelity of the evoked signal to the input stimulus in rTg4510 neurons. Together, these data suggest that loss of synapses, changes in receptor expression and dendritic restructuring may lead to alterations in synaptic integration at a network level. Understanding these compensatory processes could identify targets to help delay symptomatic onset of dementia.

## Teaser

Meftah *et al*. report alterations to synaptic and dendrite properties in the rTg4510 mouse model of tauopathy associated with disrupted synaptic integration *in vivo*. Therefore, disrupted synaptic and network integration may be early markers of synapse loss in neurodegenerative tauopathies.

## Graphical Abstract

**Graphical Abstract:**
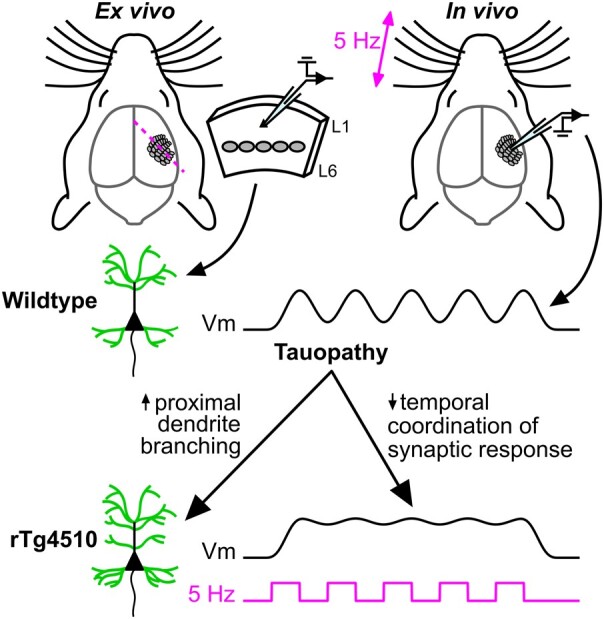

## Introduction

Tauopathies are a group of progressive neurodegenerative diseases, termed such due to their association with abnormal intracellular accumulation of tau protein.^1,2^ Cognitive decline in these diseases best correlates with synaptic degeneration, highlighting the synapse as a vulnerable and central target of tauopathy.^3-5^ Mislocalization of aggregated tau to synaptic terminals is suggested to be an early pathological process that alters synaptic function and structure, eventually leading to synapse loss.^6-12^ Multiple models of acute^12^ and chronic tauopathy show synaptic dysfunction and/or degeneration, suggesting tauopathy is sufficient to induce synaptic changes.^13-15^

Excitatory neurotransmission is thought to be particularly vulnerable to tauopathy, evidenced by the loss of glutamatergic receptor expression in post-mortem brain tissue from patients.^16-18^ NMDA receptors (NMDAR) are heavily implicated in tauopathy pathogenesis, potentially via direct interactions between tau, NMDARs and Fyn kinase that cause NMDAR-mediated excitotoxicity.^19-22^ NMDAR subunit expression is also reduced in chronic models of tauopathy, such as the rTg4510 mouse.^23^ Other alterations in glutamatergic receptors, such as down- and up-regulation of AMPA receptor (AMPAR) subunit expression, have also been observed in chronic tau overexpression models when characterized at distinct phases of the disease.^23-27^ Cultured neurons from the rTg4510 mouse model of tauopathy show tau overexpression leads to mislocalization of AMPARs at synapses with an accompanying loss of synaptic function.^28^ This dysregulation could lead to deficits in synaptic transmission and plasticity that have been observed in rTg4510 mice at a neurodegenerative phase of tauopathy.^29-31^ Altogether, mouse models of chronic tauopathy show variable alterations in glutamatergic synaptic function, with increased, decreased and unchanged excitatory neurotransmission observed.^24-27,29^ Additionally, synaptic transmission is disrupted by acute infusion of tau oligomers into hippocampal pyramidal neurons during whole-cell electrophysiological recordings, while expression of mutant tau at presynaptic terminals can impair vesicular release during repetitive stimulation.^30,31^ Therefore, it is likely that tauopathy leads to physiological disruption at synapses, but there is a lack of evidence linking expression of synaptic proteins and receptors to function, and how these effects may be modulated by disease progression.

In addition to synapse loss, there is evidence that changes at the level of the dendrite occur to compensate for altered synaptic input in tauopathy.^32,33^ In advanced Alzheimer’s disease, changes in dendritic structure, and in particular dendritic atrophy, have been observed in post-mortem brain tissue.^34,35^ In mouse models of tauopathy, there are varying dendritic phenotypes; both increased and decreased dendritic complexity have been reported, likely reflecting changes across the progression of tauopathy and differences in tau isoform expression between models.^26,36,37^ These changes in dendritic structure may occur prior to the onset of neurodegeneration, perhaps to compensate for the loss of synaptic inputs to specific dendrites.^36,37^ Changes in dendritic structure could alter synaptic integration within neurons, in addition to causing direct loss of input from lost synapses. Alternatively, functional preservation of specific afferent pathways has been reported in the rTg4510 mouse model of tauopathy, suggesting that pathology-driven changes in dendritic structure do not necessarily disrupt synaptic function.^27,38,39^ Rather, changes in dendritic complexity may preserve function or lead to compensatory alterations in tandem with changes in synaptic function at different disease phases.

In this study, we used rTg4510 mice to explore how tauopathy can alter the receptor content and function of cortical synapses in early and progressed tauopathy. Abnormal tau inclusions are detectable in rTg4510 mice from ∼2 months of age.^40-43^ The rTg4510 model also exhibits reductions in cortical synapse density and glutamatergic receptor expression as tauopathy develops. Specifically, cortical synapse loss emerges at ∼7–8 months of age and is pronounced by ∼8.5 months, by which time neurodegeneration is also evident.^23,40-44^ Interestingly, prior to synapse loss, we have previously identified aberrant dendritic spine stability and network activity from 5–6 months of age in the somatosensory cortex, implying that synaptic dysfunction may precede degeneration in rTg4510 mice.^44,45^ Building on this previous research, we again used rTg4510 somatosensory cortex as a neocortical model system to investigate properties related to synaptic instability and neuronal network dysfunction in early tauopathy. Experiments were performed in 5.5 and 7.5-month-old rTg4510 and littermate control mice, respectively. The 5.5-month age point was selected to model a pre-degenerative stage of cortical tauopathy that we have previously reported to coincide with the emergence of altered synapse stability and network activity in this model,^44^ while the 7.5-month age point modelled a more progressed, degenerative phase of tauopathy.^40-45^ We isolated crude synaptosomes to measure synaptic markers and glutamatergic receptor expression. Additionally, synaptic function in layer 2/3 (L2/3) somatosensory cortex pyramidal neurons was characterized using *in vitro* and *in vivo* whole-cell patch-clamp electrophysiology. Dendritic structure was examined from cells filled with biocytin during electrophysiological recordings.

## Materials and methods

### Ethical approval

Procedures were performed in accordance with the UK Animals (Scientific Procedures) Act 1986 and European Directive 2010/63/EU. Synaptosome extractions were performed at Eli Lilly and Co. and were approved by the Lilly UK Institutional Animal Care and Use Committee. Electrophysiological studies were performed at the Universities of Exeter and Bristol and were approved by their respective institutional Animal Welfare and Ethical Review Bodies.

### Animals

The bi-transgenic regulatable Tg4510 (rTg4510) mouse model of tauopathy was used for experiments.^40,41^ In this model, a responder 4510 mouse line carrying a tetracycline operon-responsive element upstream of cDNA encoding mutant human *MAPT^P301L^* on an FVB/NJ background is crossed with an activator mouse line which expresses a tetracycline-controlled transactivator under the control of the calcium/calmodulin kinase II α promoter on a 129S6 background. Experiments used male rTg4510 (TG) and wild-type (WT) littermate control mice of 5.5 months (5.5 M: total 35 WT mice, mean age 23 weeks; 36 TG mice, mean age 22.6 weeks) and 7.5 months (7.5 M: total 15 WT mice, mean age 33.6 weeks; 16 TG mice, mean age 33 weeks).^40-42,46^ Mice were bred at ENVIGO (Oxon, UK) and kindly provided by Eli Lilly & Company. Only male mice were available from this cohort. Mice were housed under standardized conditions (22 ± 2°C and 45 ± 15% humidity) on a 12-hour light/dark cycle with *ad libitum* access to food and water. For synaptosome analysis, brain homogenate from a P301S tauopathy mouse^47^ was used as a positive control. Experiments and analyses were performed blind to animal genotype.

### Synaptosome isolation

Mice were sacrificed by cervical dislocation. Brains were removed and the somatosensory cortex (+0.38 mm to −1.94 mm anteroposterior from Bregma) was isolated. Tissues were snap-frozen and stored at −80°C. Defrosted samples were sonicated for 10 sec (QSonica CL-18; 1 sec alternating on/off pulses, 40% pulse amplitude) in ice-cold buffer containing: 320 mM Sucrose, 10 mM EDTA, 50 mM Tris pH 7.4, protease inhibitor (cOmplete mini, Roche), Phosphatase Inhibitor Cocktail 2 (Sigma-Aldrich), Phosphatase Inhibitor Cocktail 3 (Sigma-Aldrich). Samples were centrifuged (Sigma 4–16KS, 12 130 rotor) at 1500×g for 20 min at 4°C. The supernatant was transferred to a new tube and centrifuged for 30 min at 16 000×g at 4°C. The supernatant was removed, and the pellet was resuspended in 50 mM Tris containing phosphatase and protease inhibitors (TPP). Protein concentration was measured using a BCA protein assay and samples were normalized to 1 mg/ml in TPP.

### Protein electrophoresis

Samples were diluted in Sample Buffer (2 × Laemmli, BioRad or 4 × NuPage LDS, Novex) containing 5% 2-mercaptoethanol, incubated in a heating block (Grant QBD2; 95°C, 5 min), loaded into a NuPage 8% Bis-Tris Midi Gel (Invitrogen) in 1 × MOPS SDS Running Buffer (Novex) and run at 150 V for 75 min (Invitrogen PowerEase 300W). Samples from different mouse ages and genotypes were distributed across gels in a randomized block design to minimize clustered variation. SeeBlue Plus 2 Prestained Standard (Invitrogen) was used as a molecular weight ladder.

Proteins were transferred by electroblotting (25 V, 80 min) onto a nitrocellulose membrane (0.45 µm pore size, Amersham Protran) soaked in NuPage transfer buffer (Novex) containing 20% methanol. Protein transfer was confirmed using Ponceau S (Sigma-Aldrich) and blots were cut into two at relevant molecular weights, as needed. Membranes were blocked in PBS containing 0.05% Tween (PBS-T) and 5% dried milk (PBS-T-M), and incubated (overnight, 4°C) with the primary antibody of interest (in PBS-T-M; see Supplementary Table 1). Membranes were then washed (3 × 10 min in PBS-T) and incubated (1 hr) at room temperature (RT, ∼20°C) with corresponding secondary antibodies (in PBS-T-M; Supplementary Table 1). Membranes were then washed (3 × 10 min in PBS-T). Proteins were incubated (5 min) with Dura or Femto substrates (ThermoScientific) and imaged using an Amersham Imager 600 (Supplementary Fig. 1). If required, membranes were stripped using a stripping buffer (ThermoScientific), and then re-incubated from the blocking stage.

### Synaptic protein analysis

Images were analysed using ImageQuantTL software (GE). Bands of interest were selected, and the background signal was subtracted using a rolling ball filter. Raw protein expression values were normalized within-lane to a housekeeper protein (GAPDH). Samples were excluded if the detected band (protein or housekeeper protein) was incomplete or visibly unclear.

### *In vitro* electrophysiology: recording

Mice were sacrificed by cervical dislocation at approximately the same time of day, brains were removed and placed into ice-cold sucrose solution containing (in mM): 189 Sucrose, 10 D-Glucose, 26 NaHCO_3_, 3 KCl, 5 MgSO_4_.7H_2_O, 0.1 CaCl_2_, 1.25 NaH_2_PO_4_, bubbled in carbogen (95% O_2_, 5% CO_2_). 300 µm thick thalamocortical slices were prepared as described by Agmon and Connors (1991) using a vibratome (Leica VT1200).^48^ Slices were stored at RT in artificial cerebrospinal fluid (aCSF) containing (in mM): 124 NaCl, 3 KCl, 24 NaHCO_3_, 2 CaCl_2_, 1.25 NaH_2_PO_4_, 1 MgSO_4_, 10 D-Glucose, bubbled in carbogen.

For recordings, slices were superfused with oxygenated aCSF (∼2 ml/min, ∼33°C) containing gabazine (5 µM; Abcam). A bipolar stimulating electrode was placed in the middle of visible barrel fields in layer 4 (L4) of somatosensory cortex. L2/3 putative pyramidal neurons in the same cortical column as the stimulating electrode were recorded using whole-cell patch clamp electrophysiology. Recording electrodes (4–7 MΩ) were filled with intracellular solution containing (in mM): 120 CsMeSO_4_, 6 NaCl, 5 QX314-Cl, 10 HEPES free acid, 10 BAPTA, 0.3, GTP.2Na salt, 4 ATP.Mg salt, 13.4 biocytin. Signals were amplified using an Axopatch 200B or Multiclamp 700A amplifier (Molecular Devices), digitized using an Axon Digidata 1550 (Molecular Devices) and recorded using pClamp v10.4 software (Molecular Devices). Signals were lowpass filtered at 5 or 10 kHz and sampled at 20 kHz.

Liquid junction potential error (−15 mV) was corrected. Series resistance was measured at the start of recordings. Evoked excitatory postsynaptic currents (EPSCs) were recorded in voltage clamp following electrical stimulation of L4 (1 ms duration). Stimulation intensity was adjusted to generate 50–150 pA EPSCs (mean: 58 pA) at a −70 mV holding potential. AMPAR and NMDAR currents were measured at holding potentials of −70 mV and +40 mV, respectively, with 10 recording sweeps performed for each condition. L689560 (NMDAR antagonist, 5 µm; Abcam) was used to confirm the NMDAR-dependency of +40 mV responses in a subset of experiments. EPSC paired-pulse ratio (PPR) was measured in voltage-clamp at −70 mV in response to the first two of six successive stimuli delivered at 5 Hz, 10 Hz or 30 Hz, every 15 sec, with 10 recording sweeps performed for each condition.

### *In vitro* electrophysiology: analysis

Analyses were performed using MATLAB or Clampfit v10.4 software. Cells with series resistance >40 MΩ (>95th percentile of the distribution) were excluded. Recordings for each age group were performed separately (i.e. not interleaved) with genotype blinded for the experimenter, and we observed that the series resistance was lower in the 7.5 M age group (Generalized linear mixed model (GLMM), WT mean ± SEM: 17 ± 0.6 MΩ, TG mean ± SEM: 16 ± 0.7 MΩ) compared to 5.5 M (WT mean ± SEM: 22 ± 0.7 MΩ, TG mean ± SEM: 22 ± 0.8 MΩ; genotype effect: *P* = 0.97, age effect: $\chi_{\left(1,227\right)}^{2}$ = 17.4, *P* < 0.005, interaction: *P* = 0.37). Therefore, datasets were compared within but not between age group. Occasionally, recorded sweeps exhibited observable seizure activity (due to the inclusion of gabazine in the aCSF to isolate glutamatergic synaptic responses) and were excluded from the analysis. AMPAR responses were measured as the peak response at −70 mV. NMDAR responses were measured 50 ms post stimulation at +40 mV. NMDA:AMPA ratio was calculated by dividing the NMDAR EPSC (mean of 10 sweeps) by the AMPAR EPSC (mean of 10 sweeps). PPR was calculated by dividing the peak response of the second evoked EPSC by the peak response of the first evoked EPSC.

### Histology

After recordings, slices were fixed (overnight, 4°C) in 4% paraformaldehyde in 0.1 M PBS, transferred to 0.1 M phosphate buffer (PB) and stored at 4°C. Antigen retrieval was performed by incubating slices in dH2O at 78°C for 25 min. After cooling (35 min), slices were washed in 0.1 M PB (3 × 5 min) and 0.1 M PBS (3 × 5 min). Slices were first blocked (30 min, RT) in 100 mM glycine in 0.1 M PBS containing 0.3% Triton-X and 0.05% sodium azide (PBS-T-A), and then blocked (1 hr, RT) in 0.5% normal goat serum (Vector Labs) in PBS-T-A. Slices were then incubated (3 days, 4°C) in MC-1 primary antibody^49^ (1:1000 in PBS-T-A). Slices were washed (3 × 5 min) in 0.1 M PBS and incubated (overnight, 4°C) with goat anti-mouse Alexa Fluor 594 IgG secondary antibody and anti-avidin Alexa Fluor 488 conjugate (both 1:1000 in PBS-T-A; ThermoFisher). Slices were washed (4 × 30 min) in 0.1 M PBS, rinsed (5 min) in dH2O and mounted using VectaShield (Vector Labs). Slices were imaged using two-photon (Scientifica) or confocal (Lecia SP8) microscopy. Z-stacks (0.5 µm optical slice) were taken of neuronal structure and analysed using Simple Neurite Tracer and Sholl analysis (5 µm bins) plugins in Fiji software.^50,51^

### *In vivo* electrophysiology: recordings

Experimental surgeries were all performed at a similar time of day. Mice were anaesthetized using isoflurane (induction: 3–4%, maintenance: 1–2%) and fixed in a custom stereotaxic frame with a removable baseplate. Body temperature was maintained at 37°C using a homeothermic blanket. An incision was made in the scalp and a head-fixation bar was affixed to the occipital skull plate. A dental cement well was made around somatosensory cortex and filled with aCSF containing (in mM): 135 NaCl, 5 KCl, 5 HEPES free acid, 1.8 CaCl_2_, 1 MgCl_2_. A small craniotomy was drilled at coordinates (from Bregma): + 1.3 mm anteroposterior, −3.4 mm mediolateral and the skull flap and dura were removed under aCSF. Mice received a subcutaneous dose of urethane (1.5–2 mg/kg) to maintain anaesthesia, and isoflurane was slowly withdrawn.

Mice were transferred via the baseplate to a two-photon microscope (Scientifica VivoScope) equipped with a Ti:Sapphire laser (MaiTai, Spectra-Physics). The whiskers contralateral to the recording hemisphere were bundled using Vaseline and a custom stimulator (1 mm aperture attached to a pneumatic picopump; World Precision Instruments) was positioned ∼5 mm from the whisker bundle. The brain surface was visualized under white light, and a glass recording electrode (4–10 MΩ) was inserted into the brain. The electrode internal solution contained (in mM): 135 K-Gluconate, 5 NaCl, 10 HEPES free acid, 0.2 EGTA, 0.3 GTP-Na salt, 4 ATP-Mg salt, 6.7 biocytin, 0.025 Alexa Fluor 594 Hydrazide. Two-photon imaging was used to target patch-clamp recordings to putative L2/3 pyramidal neurons using the shadow patch method.^52,53^ Some recordings were performed with MK801 (NMDAR antagonist, 1 mM; Hello Bio) in the intracellular solution. Signals were amplified using a Multiclamp 700A, digitized using a Power 1401 (CED) and recorded using Signal v5.07 software (CED). Raw signals were lowpass filtered at 10 kHz and sampled at 50 kHz. Supplementary doses of urethane were given to maintain a stable anaesthetic plane throughout recordings.

Recordings were not corrected for liquid junction potential error and bridge balance was not applied. After achieving a whole-cell recording configuration, the series resistance was measured in voltage clamp. Then, 3 minutes gap-free recording was performed in current-clamp (I = 0). Air puffs (10 mBar, 100 ms duration) were delivered to stimulate the whiskers (5 Hz, 1 s) in the rostral-caudal direction, every 30 sec, for 15 minutes. Membrane voltage (*V*_m_) was recorded throughout stimulation in current clamp (*I* = 0). Series resistance was re-checked before and after whisker stimulation.

### *In vivo* electrophysiology: analysis

Neurons were excluded if Vm exceeded 0 mV for >1 sec, if series resistance >100 MΩ, or if *V*_m_ was recorded <6 minutes during whisker stimulation. The series resistance was consistent between groups (mean ± SEM: WT control, 48.6 ± 1.8 MΩ; WT MK801, 49.8 ± 3.4 MΩ; TG control, 51.6 ± 3.7 MΩ; TG MK801, 44.5 ± 3 MΩ) (GLMM, Genotype *P* = 0.43, MK801 *P* = 0.1, Genotype*MK801 *P* = 0.16). To isolate synaptic responses to whisker stimulation, action potentials (AP) were removed by normalizing values of Vm exceeding AP threshold (d*V*_m_/d*t* >30 mV/ms) to the AP threshold.^54,55^ Responses were split into those occurring during Up states or Down states. Down states were defined as epochs of hyperpolarization with Vm range <3 mV in the 50 ms preceding whisker stimulation.^56^ Signals not meeting these criteria were classified as Up states.^56^ Up and Down state responses were normalized to the mean membrane potential (Vm) in the first 5 ms post stimulation.^56,57^ Synaptic responses to the first whisker stimulus and 5 Hz train were analysed. For the first stimuli, the peak response was the max Vm within 0–40 ms post-stimulus onset; the secondary postsynaptic potential (PSP) component was the mean Vm between 50 and 100 ms post-stimulus onset. For the 5 Hz train, the area under the curve (AUC) from stimulus onset (0 ms) to 200 ms after the last stimulus was calculated to measure the total response envelope. The cross-correlation was calculated between the stimulus train and Z-scored Vm during Down states.

### Statistical analysis

Sample sizes were estimated based on the observed effect sizes of synaptic phenotypes in our prior studies of rTg4510 mice.^29,44^ Datasets were analysed using generalized linear mixed models (GLMMs) using R (v3.5.1) with RStudio (v1.1.456). Data were visually inspected to identify appropriate distributions, link functions and factors. GLMMs included random factors to control for statistical bias and pseudo-replication.^58-60^ The intraclass correlation coefficient (ICC) evaluated whether random factors (e.g. animal, gel blot) significantly affected variance. Where ICC < 0.1 (0–1 scale), random factors were removed to avoid overfitting. The data dispersion, homogeneity of variance, Akaike information criterion and normality of residuals were used to evaluate GLMM fit. Statistical significance (*P* < 0.05) of factors in the GLMM was determined using goodness-of-fit chi squared (χ2) tests. *Post hoc* tests were performed using Tukey-Kramer multiple comparisons adjustment. Descriptive statistics are reported as mean ± SEM. Data are typically presented as box plots: squares denote the mean; boxes define ± standard error of the mean (SEM); horizontal lines denote the median; dots plot individual samples. Line graphs plot mean ± SEM.

## Results

### Reduced synaptic protein expression in the somatosensory cortex of rTg4510 mice at 5.5 and 7.5 M of age

**Figure 1:**
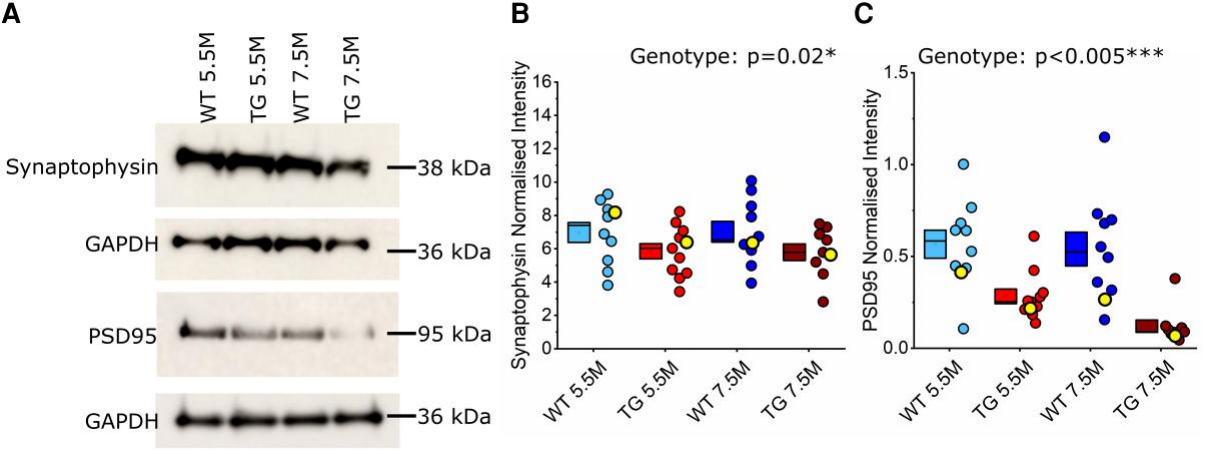
**Expression of the synaptic proteins synaptophysin and PSD95 was reduced in the somatosensory cortex in 5.5 and 7.5 M rTg4510 mice.** (**A**) Representative western blots illustrating synaptophysin and PSD95 expression in the somatosensory cortex of wild-type (WT) and rTg4510 (TG) animals at 5.5 and 7.5 months (M). The detected protein marker for the blot is annotated along the left-hand side. Annotated on the right-hand side is the corresponding molecular weight of the protein. Expression levels for each protein were normalized to the corresponding GAPDH band (below). (**B** and **C**) Box and dot plots showing quantification of each synaptic protein. Each point represents one animal (isolated somatosensory cortex). Yellow highlighted points mark the samples shown in the representative western blots in panel **A**. Statistics: Generalized linear mixed model (variable∼genotype*age + (1|gel number)), main significant effects labelled on the graph, with statistical significance denoted by asterisks (*P* < 0.05*, *P* < 0.005***). (**B**) Quantification of synaptophysin protein expression normalized to GAPDH. 5.5 M: *N* = 10 WT, 11 TG; 7.5 M: *N* = 10 WT, 9 TG (2 exclusions). (**C**) Quantification of PSD95 protein expression, normalized to GAPDH. 5.5 M: *N* = 10 WT, 11 TG; 7.5 M: *N* = 10 WT, 9 TG (2 exclusions).

To assess the effect of pathological tau on levels of synaptic proteins, crude synaptosomes were isolated from the somatosensory cortex of TG and WT littermate mice at two different ages: 5.5 M (10 WT, 11 TG) and 7.5 M (10 WT, 11 TG), respectively. Protein expression levels in synaptosomes were quantified by western blot. The presynaptic protein synaptophysin was significantly reduced in TG synaptosomes compared to WT ($\chi_{\left(1,42\right)}^{2}$ = 4.73, *P* = 0.02*, ICC = 0.53), with no age effect (*P* = 0.64) or interaction between genotype and age (*P* = 0.58; Fig. 1A and B). The excitatory postsynaptic scaffold protein, PSD95, was also significantly reduced in TG synaptosomes (genotype: $\chi_{\left(1,40\right)}^{2}$ = 14.13, *P* < 0.005***, ICC = 0.39), with no statistically significant age effect (age: $\chi_{\left(1,40\right)}^{2}$ = 3.75, *P* = 0.053, ICC = 0.39; Fig. 1A and C) and no interaction between genotype and age (*P* = 0.31). The reduced expression of synaptophysin and PSD95 in TG samples suggests synapse weakening and/or loss is an early feature of tauopathy, with postsynaptic structures exhibiting greater vulnerability.

### Reduced glutamatergic receptor expression in rTg4510 mice at 5.5 and 7.5 M of age

**Figure 2:**
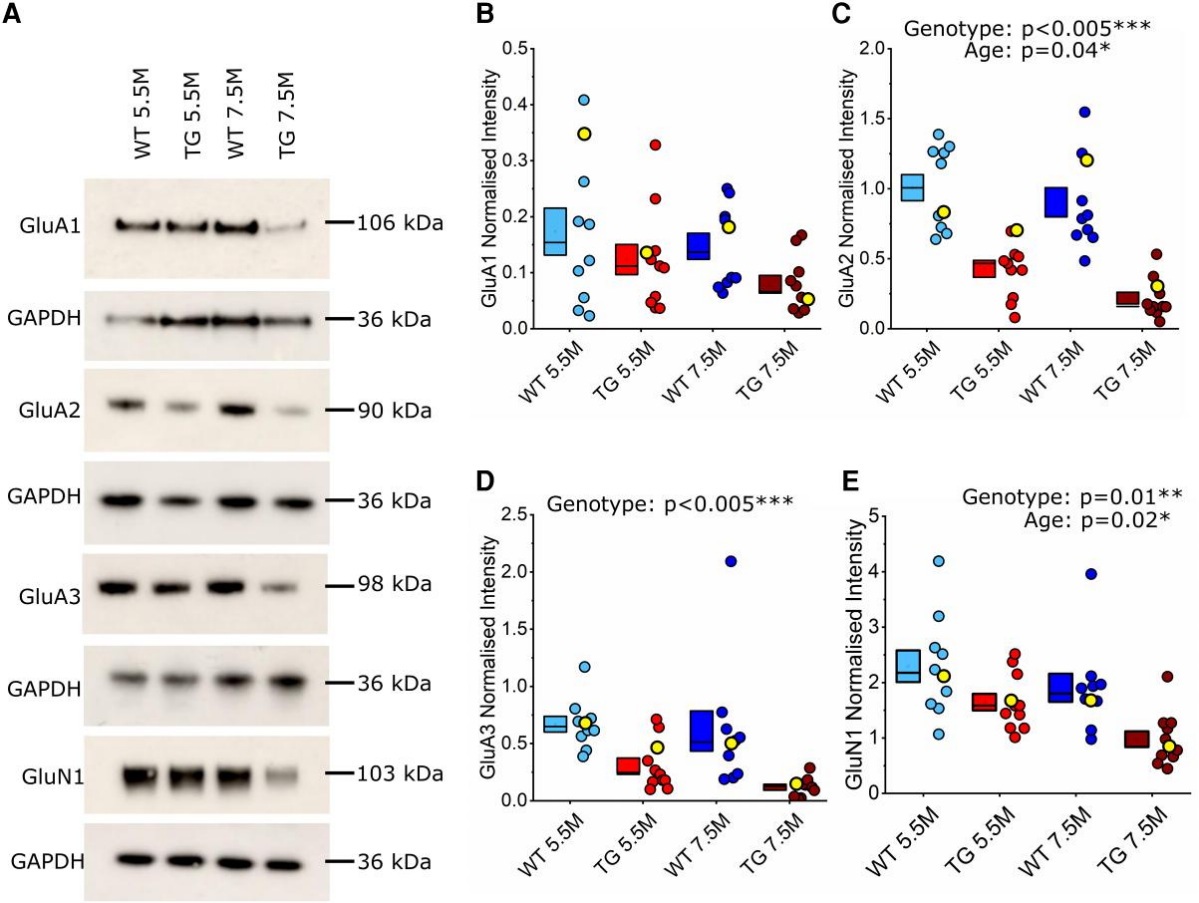
**AMPAR (GluA2 and GluA3) and NMDAR (GluN1) subunit expression was reduced in the somatosensory cortex in 5.5 and 7.5 M rTg4510 mice.** (**A**) Representative western blots of GluA1-3 and GluN1 expression in the somatosensory cortex of wild-type (WT) and rTg4510 (TG) mice at 5.5 and 7.5 months (M). The detected protein marker for the blot is annotated on the left-hand side. Annotated on the right-hand side is the corresponding molecular weight of the protein. Expression levels for each protein were normalized to the corresponding GAPDH band (below). (**B**–**E**) Box and dot plots showing quantification of each synaptic protein. Each point represents one animal (isolated somatosensory cortex). Yellow highlighted points mark the samples shown in the representative western blots in panel **A**. Statistics: Generalized linear mixed model (variable∼genotype*age + (1|gel number)), main significant effects labelled on the graph, with statistical significance denoted by asterisks (*P* < 0.05*, *P* < 0.005***). (**B**) Quantification of GluA1 expression, normalized to GAPDH. 5.5 M: *N* = 10 WT, 11 TG; 7.5 M: *N* = 10 WT, 10 TG (1 exclusion). (**C**) Quantification of GluA2 expression, normalized to GAPDH. 5.5 M: *N* = 9 WT (1 exclusion), 11 TG; 7.5 M: *N* = 10 WT, 9 TG (2 exclusions). (**D**) Quantification of GluA3 expression, normalized to GAPDH. 5.5 M: *N* = 10 WT, 11 TG; 7.5 M: 10 WT, 10 TG (1 exclusion). (**E**) Quantification of GluN1 expression, normalized to GAPDH. 5.5 M: *N* = 10 WT, 11 TG; 7.5 M: *N* = 10 WT, 11 TG.

Somatosensory cortex synaptosomes were assessed for the expression of the AMPAR subunits GluA1, GluA2 and GluA3, as these are the most prevalent AMPAR subunits in the adult rodent brain.^61-63^ There was no significant effect of genotype (*P* = 0.26), age (*P* = 0.11) or interaction between genotype and age (*P* = 0.44) on GluA1 expression (Fig. 2A and B). In contrast, there was a substantial reduction in GluA2 ($\chi_{\left(1,42\right)}^{2}$ = 29.1, *P* < 0.005***, ICC = 0.06; Fig. 2A and C) and GluA3 expression ($\chi_{\left(1,41\right)}^{2}$ = 9, *P* < 0.005***, ICC = 0.25; Fig. 2A and D) within TG synaptosomes compared to WT. This result mirrors the loss of PSD95 observed in TG mice; fewer postsynapses or less postsynaptic scaffolding may lead to fewer postsynaptic AMPARs. There was also a significant age-dependent decrease of GluA2 expression ($\chi_{\left(1,42\right)}^{2}$ = 4.33, *P* = 0.04*, ICC = 0.06), which was not seen for GluA3 (*P* = 0.15). There was no interaction between genotype and age for either GluA2 (*P* = 0.43) or GluA3 (*P* = 0.5) expression. GluN1 subunit expression was also quantified to assess NMDAR expression within synaptosomes. There was a significant effect of genotype ($\chi_{\left(1,42\right)}^{2}$ = 6.27, *P* = 0.01**, ICC 0.27) and age ($\chi_{\left(1,42\right)}^{2}$ = 5.72, *P* = 0.02*, ICC = 0.27) on the expression of GluN1, with no interaction between genotype and age (*P* = 0.62; Fig. 2A and E). Overall, this suggests that loss of the synaptic proteins synaptophysin and PSD95 is complemented by decreased AMPAR and NMDAR expression in TG mice at both 5.5 and 7.5 M ages.

### *In vitro* assessment of synaptic function: paired-pulse ratio was unaltered in rTg4510 mice

To assess cortical synaptic function, we performed whole-cell patch clamp recordings of L2/3 pyramidal neurons in somatosensory cortex brain slices from TG and WT littermate mice at 5.5 M & 7.5 M. Short-term synaptic plasticity, which depends on presynaptic release probability, was assessed by measuring the PPR of EPSCs evoked in response to electrical stimulation of L4 to L2/3 synapses. PPRs were measured in response to stimuli delivered at 5, 10 and 30 Hz, respectively. These stimulation frequencies span the theta and gamma band ranges, which are associated with the temporal organization of somatosensory cortex synaptic innervation *in vivo*.^64-68^

**Figure 3:**
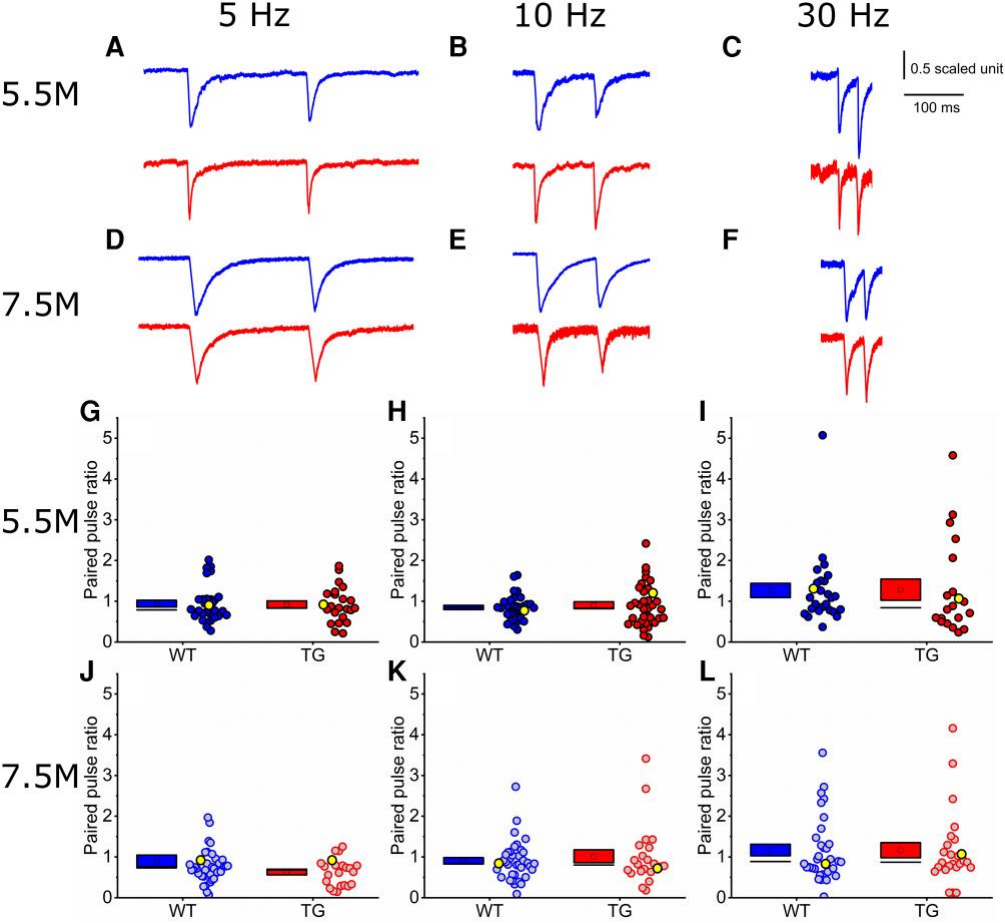
**Paired pulse ratios were similar between wild-type and rTg4510 neurons at both 5.5 and 7.5 M age points, respectively.** (**A**–**F**) Normalized sample traces for both 5.5 and 7.5 months (M) wild-type (WT; blue) and rTg4510 (TG; red) excitatory postsynaptic currents (EPSCs) (5.5 M, **A**–**C**; 7.5 M, **D**–**F**) to paired stimuli delivered at 5 Hz (**A** and **D**), 10 Hz (**B** and **E**) and 30 Hz (**C** and **F**), respectively. Traces were normalized to the amplitude of the first response. (**G**–**L**) EPSC paired-pulse ratios (PPRs) for both 5.5 and 7.5 M WT and TG neurons (5.5 M, **G**–**I**; 7.5 M, **J**–**L**) at 5 Hz (**G** and **J**), 10 Hz (**H** and **K**) and 30 Hz (**I** and **L**) stimulation frequencies. PPR values >1 suggest paired-pulse facilitation of the EPSC; PPR values <1 suggest paired-pulse depression. Note that the mean PPR is typically <1 for synaptic responses to 5 and 10 Hz stimulation, and >1 for synaptic responses to 30 Hz stimulation. Each data point corresponds to one recorded cell. Yellow highlighted points mark the representative traces shown in panels **A**–**F**. Statistics: Generalized linear mixed model (variable∼genotype*age + (1|animal number)), no significant differences observed. 5.5 M: 5 Hz: *N* = 29 (6 exclusions) WT cells (9 mice), 27 (7 exclusions) TG cells (8 mice); 10 Hz: *N* = 29 (2 exclusions) WT cells (9 mice), 23 (5 exclusions) TG cells (8 mice); 30 Hz: *N* = 26 (5 exclusions) WT cells (9 mice), 20 (6 exclusions) TG cells (8 mice). 7.5M: 5 Hz: *N* = 38 (5 exclusions) WT cells (5 mice), 22 (12 exclusions) TG cells (5 mice); 10 Hz: *N* = 34 (7 exclusions) WT cells (5 mice), 20 (9 exclusions) TG cells (5 mice); 30 Hz: *N* = 27 (13 exclusions) WT cells (5 mice), 22 (6 exclusions) TG cells (5 mice).

For the 5.5 M age group (29 WT neurons, 8 animals; 27 TG neurons, 9 animals), lower stimulation frequencies (5 and 10 Hz, respectively) produced a paired-pulse depression of EPSC amplitude in both WT and TG neurons (5 Hz: PPR of 0.85 ± 0.05 in WT cells and 0.9 ± 0.09 in TG cells, genotype effect *P* = 0.36, Fig. 3A and G; 10 Hz: 0.94 ± 0.08 in WT cells and 0.92 ± 0.09 in TG cells, genotype effect *P* = 0.84, Fig. 3B and H). At 30 Hz, paired stimuli drove a facilitating PPR that was similar in WT and TG neurons (1.26 ± 0.17 in WT cells and 1.28 ± 0.26 in TG cells, genotype effect *P* = 0.88, Fig. 3C and I).

In 7.5 M animals (38 WT neurons, 19 TG neurons, 5 animals/genotype), lower stimulation frequencies (5 and 10 Hz, respectively) produced similar responses to those in the 5.5 M cohort. 5 Hz stimulation produced a depressing PPR, while 10 Hz stimulation produced a depressing PPR for the WT group and a small facilitating PPR for the TG group (5 Hz: PPR of 0.9 ± 0.14 in WT cells and 0.63 ± 0.08 in TG cells, genotype effect *P* = 0.21, Fig. 3D and J; 10 Hz: PPR of 0.9 ± 0.09 in WT cells and 1.05 ± 0.17 in TG cells, genotype effect *P* = 0.89, Fig. 3E and K). At 30 Hz, the mean PPR was facilitating in both WT and TG neurons (1.16 ± 0.16 in WT cells and 1.26 ± 0.2 in TG cells, genotype effect *P* = 0.95, Fig. 3F and L).

Overall, there was no significant effect of genotype on short-term synaptic plasticity measures in either the 5.5 M or 7.5 M age groups. This suggests that, in this model of tauopathy, there are no apparent changes to neurotransmitter release probability at L4 to L2/3 synapses, suggesting that presynaptic release is unaltered by tauopathy at this disease phase in the rTg4510 model.

### *In vitro* assessment of synaptic function: NMDA:AMPA receptor response ratio was unaltered in rTg4510 mice

**Figure 4:**
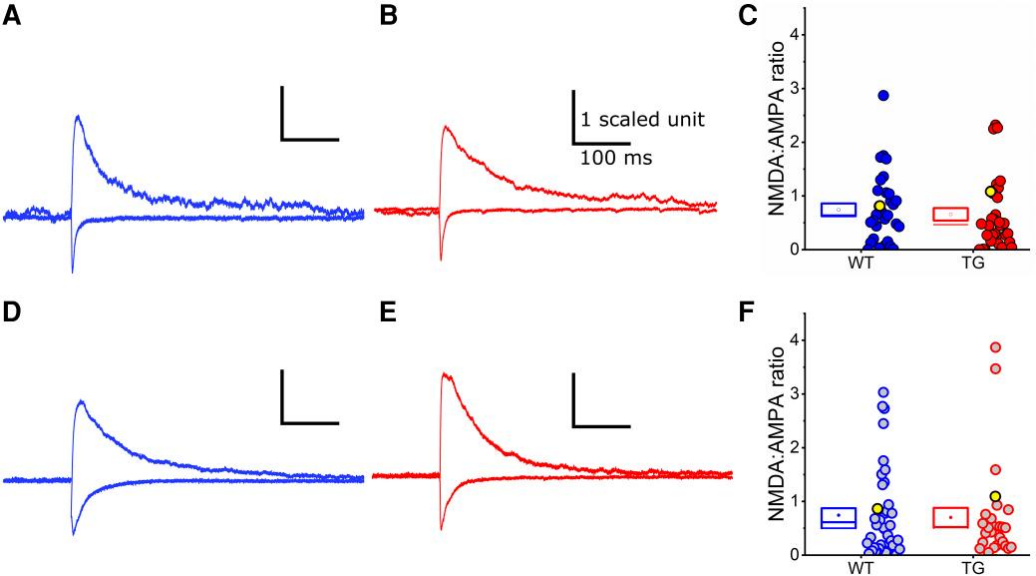
**The NMDA:AMPA receptor response ratio was similar between wild-type and rTg4510 neurons at both 5.5 and 7.5 M age points, respectively.** (**A, B, D, E**) Example waveforms of NMDAR and AMPAR excitatory postsynaptic currents (EPSCs), normalized to the AMPAR peak component (wild-type (WT): blue, **A** and **D**; rTg4510 (TG): red, **B** and **E**). The stimulus artefact has been removed for visualization. **A** and **B**) EPSC waveforms from WT and TG neurons at 5.5 months (M) of age. (**C**) Box and dot plots showing pooled NMDA:AMPA receptor ratios at 5.5 M. Dots correspond to individual neurons. Yellow highlighted points mark the representative traces shown in panels **A** and **B**. *N* = 39 (8 exclusions) WT neurons (10 mice), 33 (8 exclusions) TG neurons (10 mice). (**D** and **E**) EPSC waveforms from WT and TG neurons at 7.5 M of age. (**F**) Box and dot plots showing pooled NMDA:AMPA receptor ratios at 7.5 M. Dots correspond to individual neurons. *N* = 42 (8 exclusions) WT neurons (5 mice), 30 (11 exclusions) TG neurons (5 mice). (**C** and **F**) Statistics: Generalized linear mixed model (variable∼genotype*age + (1|animal number)), no significant differences observed.

Decreased AMPAR and NMDAR subunit expression (Fig. 2) could cause changes in the synaptic responses mediated by these receptors. Therefore, we tested the functional receptor content of L4 to L2/3 synapses by measuring the NMDA:AMPA receptor EPSC ratio. There was no significant effect of genotype at 5.5 M ($\chi_{\left(1,63\right)}^{2}$ = 0.1, *P* = 0.75; Fig. 4A–C; 39 WT neurons, 33 TG neurons, 10 animals/genotype) or at 7.5 M ($\chi_{\left(1,72\right)}^{2}$ = 0.12, *P* = 0.74; Fig. 4D–F; 42 WT neurons, 30 TG neurons, 5 animals/genotype). This suggests that L4 to L2/3 synapses maintain relative levels of functional postsynaptic glutamate receptors despite putative synapse loss during progressive tauopathy.

### Cortical pyramidal neurons exhibited increased dendritic branching proximal to the soma in 5.5 M rTg4510 mice

**Figure 5:**
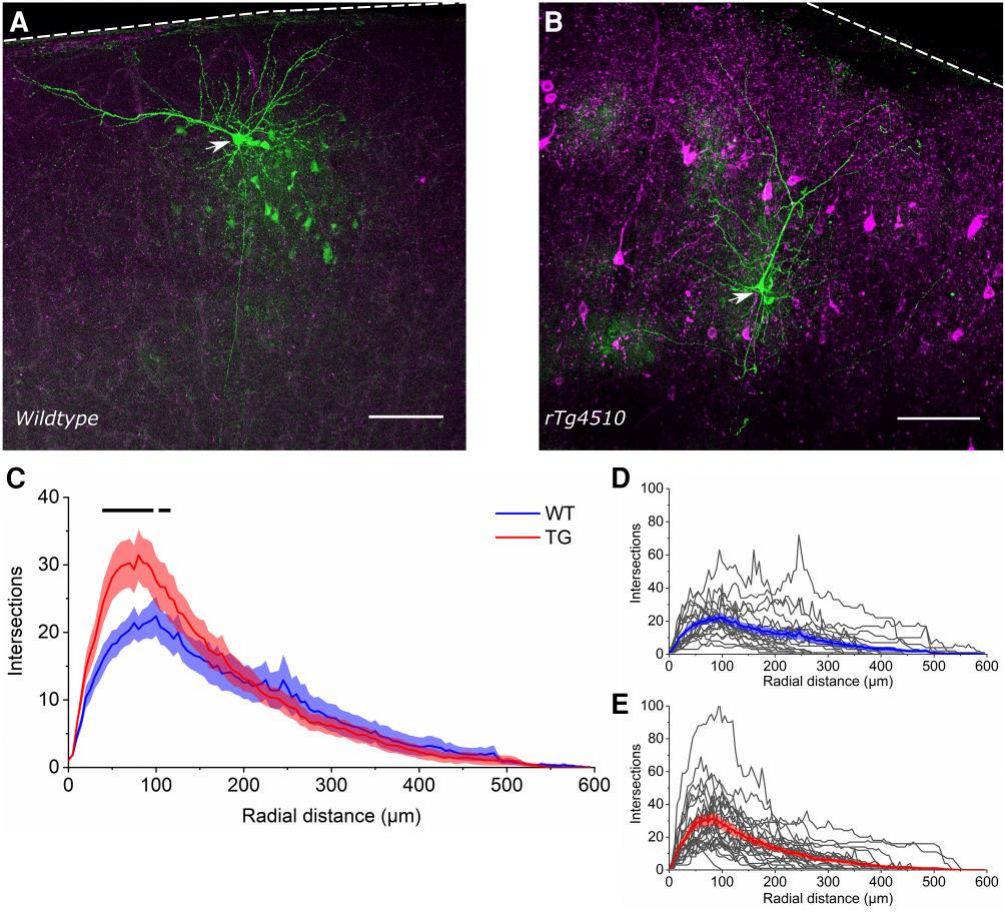
**Restructuring of proximal dendrites in rTg4510 cortical pyramidal neurons at 5.5 M.** (**A** and **B**) Photomicrographs of representative morphologically recovered wild-type (WT) and rTg4510 (TG) neurons (green, Alexa Fluor 488). The soma of each recovered neuron is denoted by a white arrowhead. Immunolabelling for conformationally altered tau (MC-1 antibody) is shown in magenta. Note that anti-tau immunolabelling is absent in WT tissue (**A**) but prolific in TG tissue (**B**). Scale bar: 100 µm. The dashed white line denotes the location of the pia. (**C**) Sholl plot of the average number of dendrite intersections per radial distance from the soma for WT (blue) and TG (red) cortical pyramidal neurons. The graph plots the mean (thick lines) ± SEM (shading). The black line above the plot denotes statistical significance between WT and TG at *P* < 0.05. Statistics: Generalized linear mixed model (variable∼genotype*intersections + (1|animal number/cell number)) with statistically significant *P* values (per radial distance, in µm) as follows: 35, *P* = 0.02; 40, *P* = 0.005; 45, *P* < 0.005; 50, *P* < 0.005; 55, *P* < 0.005; 60, *P* < 0.005; 65, *P* < 0.005; 70; *P* < 0.005; 75, *P* = 0.008; 80, *P* < 0.005; 85, *P* < 0.005; 90, *P* < 0.005 95, *P* = 0.01; 105, *P* = 0.05; 110, *P* = 0.03. (**D** and **E**) Sholl plots for all morphologically reconstructed WT (**D**) and TG neurons (**E**). Thick lines represent the mean. *N* = 21 WT neurons (10 mice), 26 TG neurons (13 mice).

Since decreased expression of synaptic proteins in somatosensory cortex (Figs 1 and 2) was not associated with changes in glutamatergic synaptic transmission at L4 to L2/3 synapses in 5.5 M TG mice (Figs 3 and 4), we assessed whether changes in neuronal structure may be present to possibly compensate for altered synapse density and/or receptor subunit composition.^27^ Sholl analysis was used to compare dendritic reconstructions of neurons from the 5.5 M age group that were labelled with biocytin during electrophysiological recordings (21 WT neurons, 10 animals; 26 TG neurons, 13 animals; Fig. 5A and B). Insufficient neurons were recovered to enable this analysis in the 7.5 M age group.

This analysis revealed a significant main effect of radius ($\chi_{\left(119,5520\right)}^{2}$ = 5715, *P* < 0.005***), but not of genotype (*P* = 0.89). There was, however, a significant interaction between genotype and radius, suggesting that dendritic branching differed between TG and WT neurons at local subregions of the dendrite ($\chi_{\left(119,5520\right)}^{2}$ = 310.4, *P* < 0.005***). Specifically, TG neurons had significantly more dendritic branch intersections in the region proximal to the soma (within 35–110 µm; Fig. 5C–E). Therefore, while we did not observe overt dysfunction of L4 to L2/3 synaptic transmission in 5.5 M TG mice, there may be altered network-level integration of synaptic input across the dendritic tree due to dendritic restructuring.

### *In vivo* assessment of synaptic function: sensory stimulation-evoked synaptic responses had an increased peak amplitude in 5.5 M rTg4510 mice

L2/3 somatosensory cortex pyramidal neurons receive differentially distributed synaptic input onto the dendritic tree, with the distal apical tuft receiving long-range and higher order inputs from distal areas while the proximal dendrites receive local L4, L2/3 and first-order thalamic inputs.^69-72^ Therefore, alterations in dendritic structure may disrupt the integration of synaptic inputs with complex spatiotemporal distribution across the dendrite. To assess this, we used *in vivo* whole-cell patch clamp recordings of L2/3 barrel (i.e. whisker somatosensory) cortex pyramidal neurons in urethane anaesthetized WT and rTg4510 mice to measure functional synaptic integration at 5.5 M (*N* = 15 animals/group). Stimulation was delivered using air puffs to generate rhythmic (5 Hz for 1 s) rostrocaudal deflections of the contralateral whiskers.

**Figure 6:**
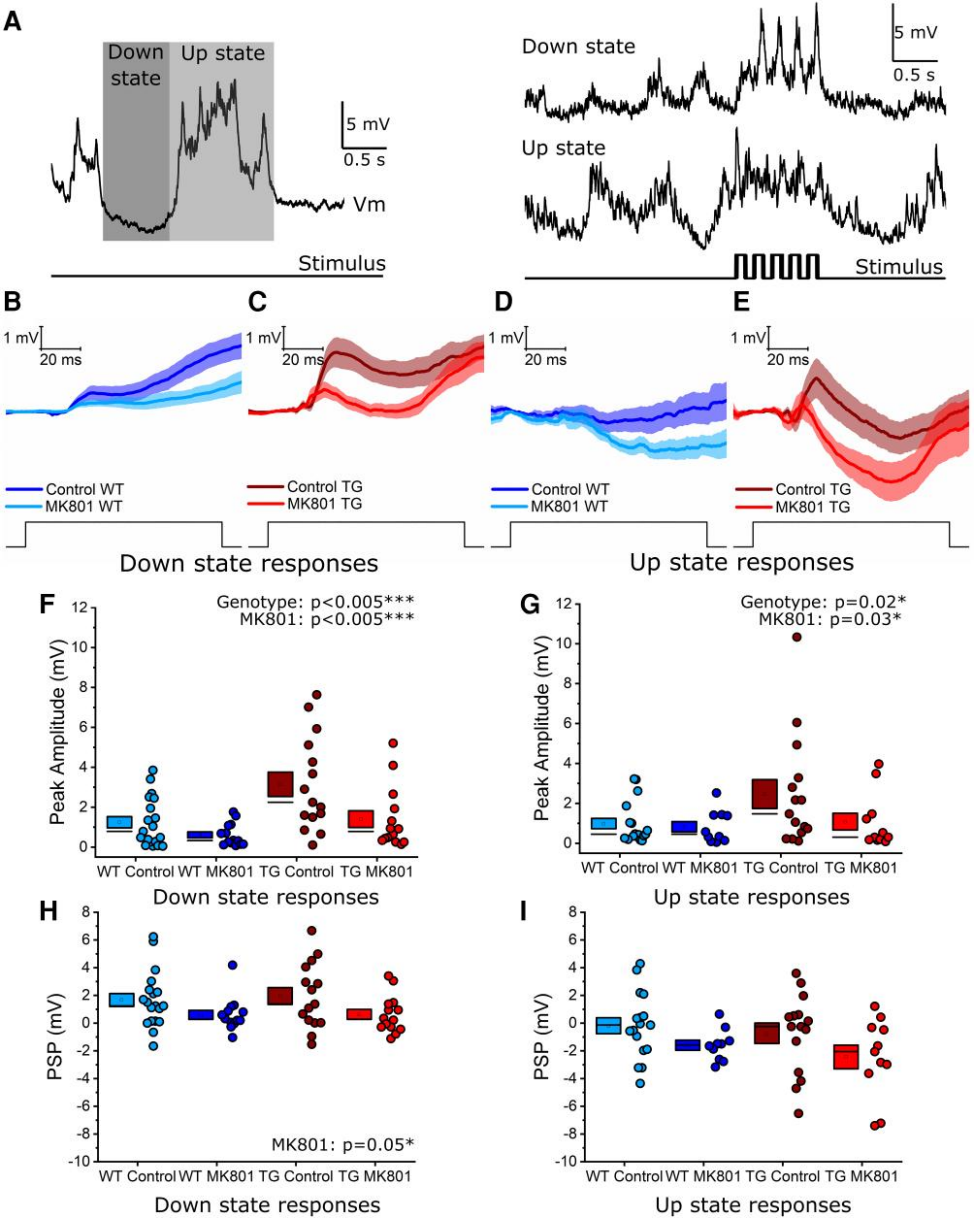
**Sensory stimulation elicited larger initial synaptic responses in 5.5 M rTg4510 cortical pyramidal neurons.** (**A**) Left: An example voltage trace showing representative Up states (light shading) and Down states (dark shading) in an *in vivo* whole-cell recording of a putative layer 2/3 somatosensory cortex pyramidal neuron. Right: An example synaptic response evoked by rhythmic (5 Hz, 1 s) whisker deflection during a Down state (*top*) and Up-state (*bottom*); the stimulus time course is plotted beneath the voltage traces. (**B** and **C**) Average synaptic responses to the first whisker deflection of the 5 Hz train during Down states in wild-type (WT) (**B**) and rTg4510 (TG) (**C**) neurons. Both control and MK801 conditions are shown. Thick lines represent the mean, with shading representing SEM. (**D** and **E**) As for panels **B** and **C** during Up states in WT (**D**) and TG (**E**) neurons. Thick lines represent the mean, with shading representing SEM. (**F** and **G**) Quantification of the initial (0–40 ms post stimulation) peak of the synaptic response evoked by whisker deflection during Down states (**F**) and Up states (**G**). (**H** and **I**) Quantification of the average postsynaptic potential (PSP) 50–100 ms after whisker deflection during Down states (**H**) and Up states (**I**). Statistics: Generalized linear mixed model (variable∼genotype*MK801 treatment + (1|animal number)), main significant effects labelled on the graph, with statistical significance denoted by asterisks (*P* < 0.05*, *P* < 0.005***). WT Control: 19 (1 exclusion) cells (12 animals), WT MK801: 14 cells (8 animals); TG Control: 15 (1 exclusion) cells (10 animals), TG MK801: 14 cells (9 animals).

Under urethane anaesthesia, neuronal Vm cycles between Up and Down states, which impacts the response to the incoming synaptic drive.^73-76^ Up–Down state oscillations were consistently observed in recordings from WT and TG neurons (Fig. 6A), so stimulus-evoked PSPs were separated based on whether the onset of whisker deflection coincided with an Up or Down state. Whisker deflection generated complex PSP waveforms combining depolarizing and hyperpolarizing phases that suggested integration of multiple excitatory and inhibitory synaptic conductances (Fig. 6B–E). First, the response to the initial whisker deflection was analysed by measuring the peak Vm immediately (0–40 ms) after whisker deflection and the average PSP between 50–100 ms post deflection, which corresponds to a period of integration of polysynaptic inputs triggered by the whisker deflection.^77^

The peak of the whisker deflection-evoked response was significantly larger in TG neurons compared to WT controls during both Down states (genotype effect: $\chi_{\left(1,62\right)}^{2}$ = 10.7, *P* < 0.005***, ICC = 0.11) and Up states (genotype effect: $\chi_{\left(1,53\right)}^{2}$ = 5.2, *P* = 0.02*, ICC = 0.23; Fig. 6F and G). Interestingly, intracellular application of the use-dependent NMDAR antagonist MK801 via the recording pipette significantly reduced the peak response during both Down and Up states (Down state MK801 effect: $\chi_{\left(1,62\right)}^{2}$ = 10.4, *P* < 0.005***; Up state MK801 effect: $\chi_{\left(1,53\right)}^{2}$ = 4.7, *P* = 0.03*; Fig. 6F and G). This shows that postsynaptic NMDARs contributed to the initial whisker deflection-evoked depolarization, but there was no difference in NMDAR contribution between WT and TG neurons in either Down (*P* = 0.91) or Up (*P* = 0.25) states.

The average PSP 50–100 ms post-whisker-deflection was not different between genotypes during Down states (*P* = 0.61) or Up states (*P* = 0.53; Fig. 6H and I). As expected, MK801 treatment affected PSP amplitude, significantly decreasing the PSP during Down states (MK801 effect: $\chi_{\left(1,62\right)}^{2}$ = 3.8, *P* = 0.046*; Fig. 6H) with no effect on Up state responses (*P* = 0.08; Fig. 6I). There was no interaction between genotype and MK801 treatment on PSP responses during Down (*P* = 0.82) or Up (*P* = 0.79) states.

### *In vivo* assessment of synaptic function: the compound synaptic charge evoked by rhythmic whisker stimulation was not altered in 5.5 M rTg4510 cortical pyramidal neurons

**Figure 7:**
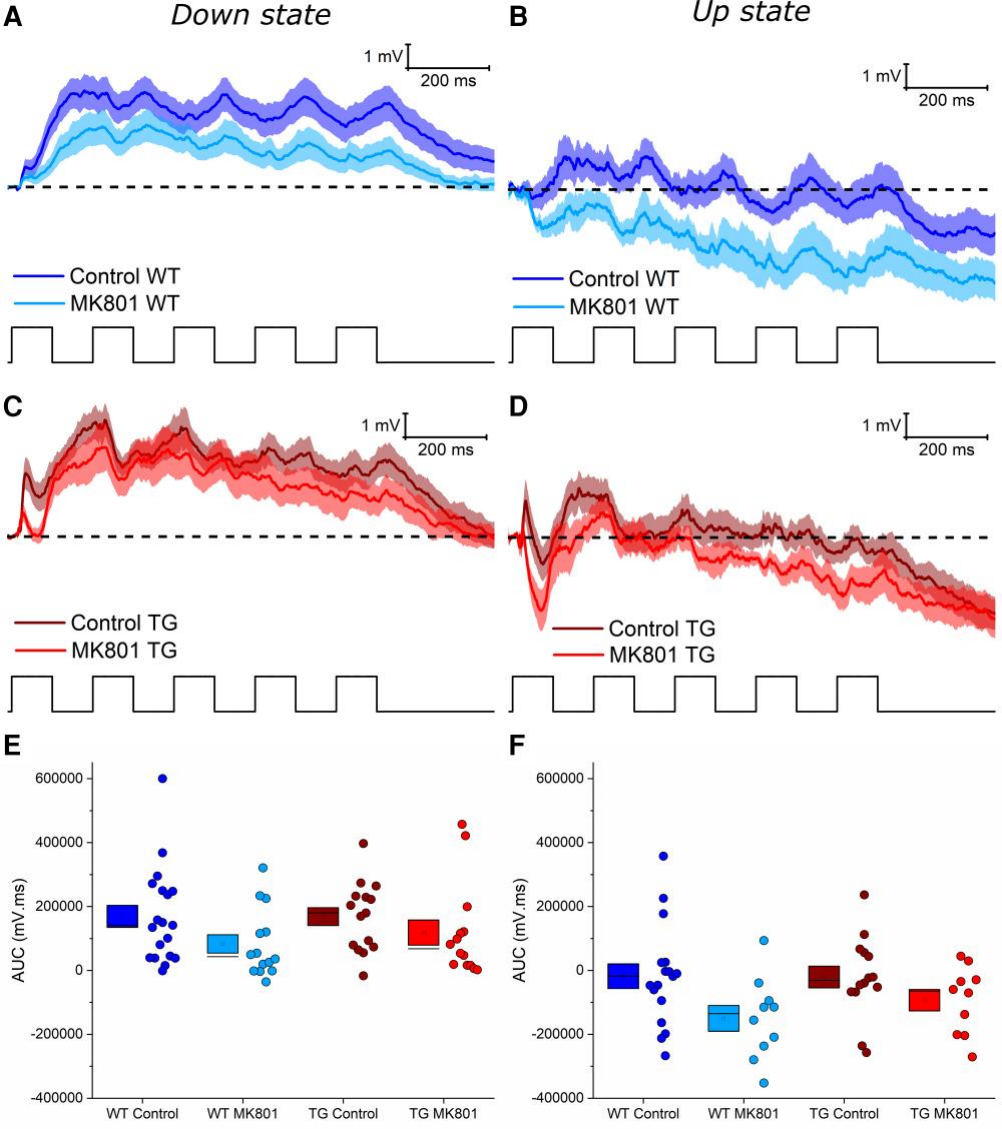
**Genotype or MK801 treatment did not alter the compound synaptic charge evoked by 5 Hz sensory stimulation in 5.5 M rTg4510 cortical pyramidal neurons.** (**A**–**D**) Average compound synaptic responses evoked by a 5 Hz, 1 s train of whisker stimuli during Down states (**A** and **C**) and Up states (**B** and **D**) for wild-type (WT) (**A** and **B**) and rTg4510 (TG) (**C** and **D**) neurons. Thick lines represent the mean, with the shading representing the SEM. Dashed lines represent the pre-stimulus membrane voltage (Vm) to which the area under the curve (AUC) was normalized. Note that trains of stimuli during Down states typically evoked net depolarization of Vm, whereas trains during Up states often caused net hyperpolarization. (**E** and **F**) Pooled data illustrating the AUC of the stimulus train-evoked compound synaptic response during Down states (**E**) and Up states (**F**) states. Statistics: Generalized linear mixed model (variable∼genotype*MK801 treatment + (1|animal number)), no significant differences observed. WT Control: 19 (1 exclusion) cells (12 animals), WT MK801: 14 cells (8 animals); TG Control: 15 (1 exclusion) cells (10 animals), TG MK801: 14 cells (9 animals).

Next, the response to 5 Hz whisker deflection was examined. This stimulus generated an envelope of synaptic activity that could be used to examine the integration of sensory drive. Thus, the area under the curve (AUC) of the entire PSP normalized to the pre-stimulus membrane potential was measured to assess overall synaptic activation (Fig. 7A–D). During Down states, there was no significant effect of genotype (*P* = 0.85), or MK801 (*P* = 0.29), and no interaction between genotype and MK801 treatment (*P* = 0.37; Fig. 7E). This was also the case for responses initiated during Up states, with no significant effect of genotype (*P* = 0.98), MK801 treatment (*P* = 0.24) or interaction between genotype and MK801 treatment (*P* = 0.35; Fig. 7F). Therefore, despite genotype- and MK801-dependent differences in the response to initial whisker deflection (Fig. 6), the compound synaptic activation evoked by a stimulus train was similar between genotypes and with MK801 treatment.

### Reduced temporal coordination of *in* vivo synaptic responses to rhythmic whisker stimulation in 5.5 M rTg4510 mice

**Figure 8:**
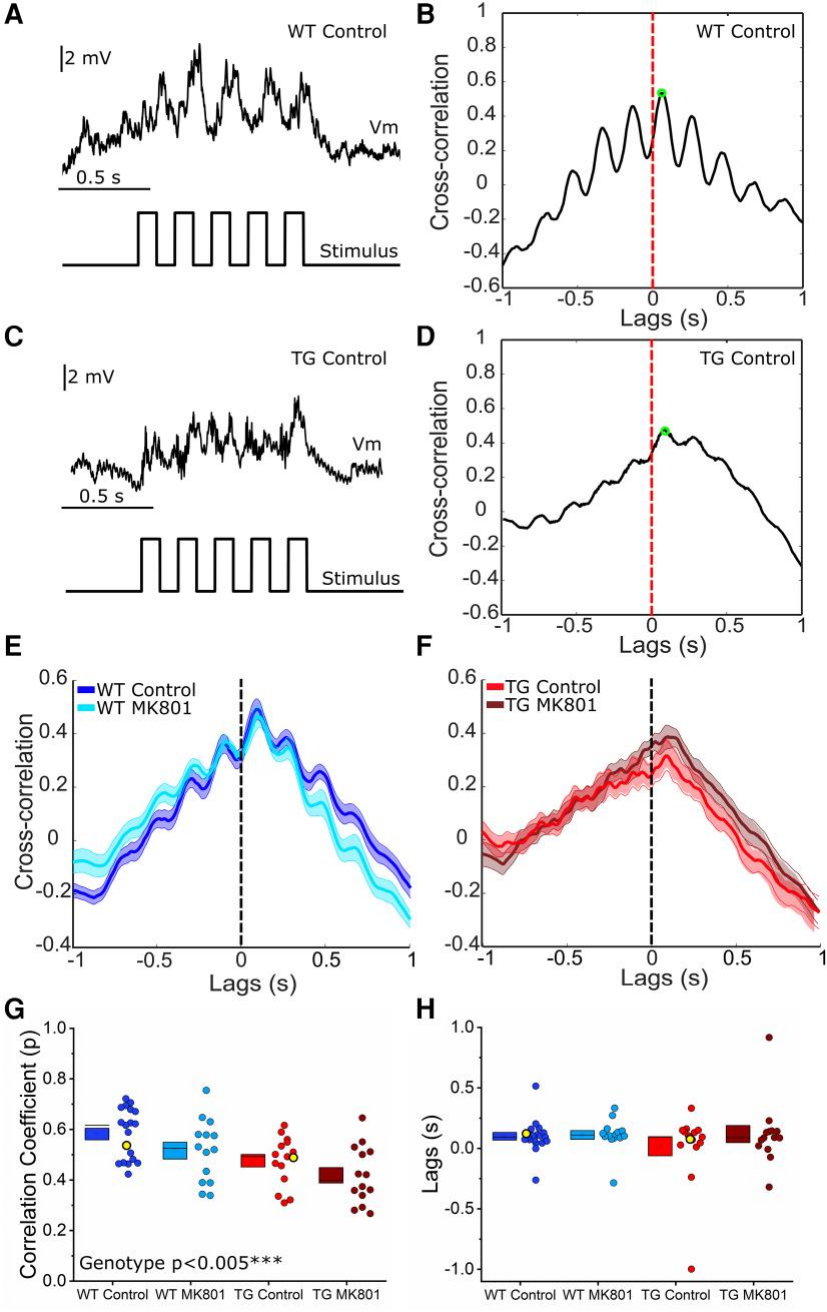
**Cross-correlation between 5 Hz sensory input and evoked synaptic responses is reduced in 5.5 M rTg4510 cortical pyramidal neurons.** (**A**–**D**) Left: Example voltage traces (*top*) illustrating the synaptic response to 5 Hz whisker stimulation (*bottom*) in wild-type (WT) (**A**) and rTg4510 (TG) (**C**) somatosensory cortex layer 2/3 pyramidal neurons. Right: Example cross-correlation between membrane voltage (Vm) and the whisker stimulation time course for WT (**B**) and TG (**D**) neurons illustrated in the left-hand panels (**A** and **C**, respectively). The peak of the cross-correlation is annotated by a green circle; the cross-correlation at 0 lags is annotated by a red dashed line. (**E** and **F**) Average cross-correlation for WT (**E**) and TG (**F**) neurons, with or without MK801 treatment. Thick lines represent the mean, with shading representing SEM. (**G**) Box and dot plots showing the peak cross-correlation coefficient for each neuron. (**H**) Box and dot plots showing the lag of the peak correlation coefficient for each neuron. Dots in **G**–**H** correspond to individual neurons. Yellow highlighted points mark the traces shown in panels **A** and **C**. WT Control: 19 cells (12 animals), WT MK801: 14 cells (8 animals); TG Control: 15 cells (10 animals), TG MK801: 14 cells (9 animals). Statistics: Generalized linear mixed model (variable∼genotype*MK801 treatment + (1|animal number)), main significant effects labelled on the graph, with statistical significance denoted by asterisks (*P* < 0.005***).

While compound synaptic activation was similar between genotypes, inspection of WT and TG responses to 5 Hz whisker deflection (Fig. 7A–D) indicated that the temporal profile of the response was altered between genotypes. Cross-correlation analysis was performed between the 5 Hz stimulus signal and the synaptic response to determine how faithfully the response followed the input pattern (Fig. 8A–D). Due to differences in driving force between Up and Down states leading to differences in the response size, and to align with previous literature on cortical synaptic responses to whisker stimulation-evoked activity, analysis was performed on Down-state responses.^75,77-79^ This revealed a clear loss of rhythmicity in TG synaptic responses compared to WT controls (peak cross-correlation: $\chi_{\left(1,62\right)}^{2}$ = 8.2, *P* < 0.005; Fig. 8E–G). Measurement of the peak correlation lags to assess whether there was a shift in stimulus-response timing revealed no genotype effect (*P* = 0.27; Fig. 8H), suggesting the peak stimulus–response correlation occurred at a similar time in WT and TG neurons. There was no effect of MK801 on the peak correlation coefficient or lags (peak: *P* = 0.16; lags: *P* = 0.23), or genotype-MK801 interaction effect (peak: *P* = 0.79; lags: *P* = 0.46), suggesting that stimulus-response decorrelation in rTg4510 neurons was not dependent on NMDAR function (Fig. 8E–H). Thus, while synaptic responses in WT neurons were faithfully temporally modulated by 5 Hz whisker deflection, encoding of this rhythmic sensory input was substantially degraded in TG neurons.

## Discussion

We have investigated perturbations to cortical synapses in the early phases of tauopathy. While at the tissue level we observed a marked loss of synaptic proteins at earlier and more progressed phases of disease in rTg4510 mice, we found subtleties in how these effects link to synaptic function and changes in neuronal morphology. Specifically, our data suggests that early tauopathy is associated with a shift in the spatial and temporal organization of synaptic inputs to cortical pyramidal neurons. As a result, the ability of these neurons to faithfully encode temporally coordinated synaptic signals is degraded, suggesting a mechanism that may underlie early symptoms of tauopathy.

A point to consider is how these results from a single rodent model that recapitulates some aspects of dementia-like pathology generalizes to human tauopathies. In particular, the rTg4510 mouse model is now known to exhibit off-target mutations around the site of MAPT^P301L^ transgene insertion that may contribute to pathological phenotypes.^80,81^ However, studies that have used doxycycline to suppress transgene expression at different developmental age points in rTg4510 mice have shown a range of tauopathy-driven cellular and synaptic phenotypes.^42,82^ Overall, the rTg4510 mouse is one of only a few well-characterized models that display robust tau pathogenesis and neurodegeneration. Nonetheless, studies examining neuronal morphology and synaptic alterations in other models of early and progressed tauopathy, such as those with mutations expressed under endogenous promoters,^83^ will be important comparators to this work. We also note that this study only investigated male rTg4510 mice. More aggressive initial development of tauopathy has been reported in female rTg4510 mice, including increased expression of hyperphosphorylated tau at 5.5 M.^84,85^ As well as comparison to other tauopathy models, to validate the pathological basis of phenotypes observed in this study it will be important to confirm these generalize to equivalently staged tauopathy in female rTg4510 mice, likely at a younger age point.

Biochemical assessment of cortical synaptosomes showed a profound decrease in PSD95—a key component of excitatory postsynapses—in rTg4510 mice (Fig. 1). The magnitude of this effect was not mirrored by synaptophysin—a key presynaptic protein—which was subtly reduced in rTg4510 mice (Fig. 1). This suggests a particular vulnerability of postsynapses compared to presynaptic compartments to tauopathy. Previous longitudinal imaging of axonal boutons and dendritic spines (synonymous with pre- and post-synaptic components, respectively) in similarly aged (5.5 M) rTg4510 mice showed increased bouton stability accompanied by increased spine turnover.^44,45^ Divergence of functional pathology in pre- and post-synaptic compartments may be a feature of early tauopathy. Within the postsynapse, PSD95 anchors glutamatergic receptors, promoting synaptic stability.^86,87^ Therefore, decreased stability of dendritic spines could be caused by lower levels of PSD95. Many studies have reported decreased levels of pre- and post-synaptic proteins and structures in advanced, degenerative stages of tauopathy in rTg4510 mice, and in other models of tauopathy.^23,37,88^ This is usually thought to reflect a generalized synapse loss that accompanies neurodegeneration. The reduced expression of PSD95 and synaptophysin we report in 5.5 M rTg4510 mice may reflect an early loss of particularly vulnerable synapses or a period of synaptic pathophysiology potentially associated with decreased synaptic stability.^44^ Our biochemical, structural and electrophysiological data suggest both occur.

We observed a substantial decrease in several AMPAR and NMDAR subunits (specifically, GluA2, GluA3 and GluN1) in somatosensory cortex samples from rTg4510 mice (Fig. 2). We were therefore surprised to find no change in the NMDA:AMPA receptor EPSC ratio in electrophysiological recordings of L4 input to L2/3 pyramidal neurons (Fig. 4). We also observed no change in PPR as a measure of short-term synaptic plasticity in this pathway, suggesting that presynaptic release is likely unaffected at these synapses in early tauopathy (Fig. 3). While our *in vitro* electrophysiological recordings did not reveal functional differences at L4 to L2/3 synapses, we note that deficits have been observed in other synaptic pathways and in other models of tauopathy, often associated with differing levels of pathology.^89^ Thus, tauopathy may differentially affect particular synaptic pathways and neuronal populations at distinct stages of disease progression.^17,18^ For example, it is possible that intracolumnar L4 to L2/3 synapses were preserved in our study, while effects on other synapses in the same cortical region caused changes in synaptic protein expression in rTg4510 mice, implying that specific subpopulations of synapses could be more vulnerable to tauopathy than others. Indeed, we observed changes to the morphology of dendrites proximal to the soma of 5.5 M rTg4510 pyramidal neurons, suggesting that tauopathy can drive alterations in specific parts of the neuron (Fig. 5). In degenerative stages of pathology in rTg4510 mice and other murine tauopathy models, there is evidence of dendritic atrophy in cortical pyramidal neurons reminiscent of that found in Alzheimer’s disease post-mortem brain tissue.^26,32,33,36,37^ Notably, however, it has been shown that some neurons appear spared from degeneration.^26^ The dendritic changes we observed in 5.5 M rTg4510 neurons were not associated with overt signs of cellular atrophy, suggesting that dendritic remodelling could represent an early homeostatic compensation for altered synaptic input. Increased branching proximal to the soma could lead to an altered balance of inputs from different anatomically distributed synaptic pathways that target specific parts of the dendritic tree.^69,70,90,91^ For example, local and sub-cortical inputs tend to cluster on basal or proximal dendrites, whereas apical dendrites tend to receive long-range inputs.^69,70,72,90,91^ If additional synapses are formed onto the more complex proximal branches of rTg4510 neurons, we would anticipate a shift in the spatial integration of synaptic input that might alter neuronal response properties.

To investigate how changes in synaptic receptor expression and dendritic structure contribute to signal processing, *in vivo* whole-cell patch clamp recordings were made during whisker stimulation to evoke physiological multimodal synaptic responses resulting from the integration of local (intracolumnar) and long-range (intercolumnar) synaptic input. These experiments revealed a functional impact of early tauopathy on synaptic encoding. Depolarization evoked by the first whisker deflection was increased in rTg4510 neurons, revealing altered synaptic input (Fig. 6). This brief depolarization is likely dominated by direct thalamocortical input, which preferentially targets the proximal basal dendrites that we found were more complex in L2/3 rTg4510 pyramidal neurons (Fig. 5).^72^ We speculate that thalamocortical axons may form additional synapses onto the more complex proximal dendrites of rTg4510 neurons, causing increased depolarization. Loss of cortical GABAergic synapses has also been reported in 5.5 M rTg4510 mice,^92^ and it is possible that decreased feedforward inhibition could contribute to this phenotype. The initial EPSP was, in part, mediated by NMDARs because its peak was decreased by MK801 (Fig. 6). It should be noted, however, that intracellular application of MK801 does not lead to a complete block of NMDARs, and so while this does not change our conclusions from this experiment, NMDARs may still have contributed to synaptic responses post MK801 application.^93^ However, we did not find any difference in NMDAR contribution between WT and rTg4510 neurons to these EPSPs.

Rhythmic (5 Hz) trains of whisker deflections generated sustained postsynaptic depolarization in WT and rTg4510 neurons (Fig. 7). However, the shape of the response to rhythmic multi-whisker stimulation was markedly different. Whereas WT neurons exhibited frequency-modulated responses to rhythmic input, this was blunted in rTg4510 neurons (Fig. 8). The PSP waveform elicited by this protocol likely reflects the integration of excitatory and inhibitory synaptic potentials arising from multiple intracolumnar and intercolumnar pathways.^56,75,77^ We speculate that changes in GABAergic inhibition onto rTg4510 neurons could potentially explain this change in the response dynamics. Notably, local GABAergic interneurons evoke inhibitory postsynaptic potentials 10–20 ms post whisker stimulation,^57^ and stimulation of multiple whiskers activates excitatory and inhibitory inputs in multiple cortical columns that contribute to the compound PSP.^75^ Deficits in synaptic protein expression—like those we have observed (Figs 1 and 2)—in GABAergic interneurons, may reduce their ability to be recruited by sensory input. Evidence to support this idea comes from observation of reduced synapse density in interneurons during early tauopathy in the rTg4510 model.^92^ Consistent with this, the timing of interneuron activity is disrupted during sharp wave-ripple oscillations in the hippocampus of rTg4510 mice,^38^ implying that disrupted timing of GABAergic input may hinder entrainment of rTg4510 neurons to rhythmic input. Therefore, alterations in coordinated synaptically-driven activity are not only observed in the somatosensory cortex and may be a generalized mechanism of early neural circuit disruption in tauopathy.

## Conclusions

Our research has identified significant reductions in glutamatergic receptor and synaptic marker expression during early tauopathy. These changes occurred alongside altered dendritic structure in cortical pyramidal neurons, which was potentially linked to changes in synaptic function, particularly entrainment of synaptic responses to rhythmic multimodal input. Our findings suggest that synaptic alterations in early tauopathy likely manifest through disrupted coordination of neural network activity. Understanding how such changes precipitate altered signal processing may be critical to preventing symptoms in tauopathy-associated dementia.

## Supplementary Material

fcae134_Supplementary_Data

## Data Availability

The data and analysis code used for this study are available from the corresponding author, upon reasonable request.
