# Supplementary material for: Synaptic alterations associated with disrupted sensory encoding in a mouse model of tauopathy
Source: Brain Commun. 2024 Apr 15;6(3):fcae134. doi: 10.1093/braincomms/fcae134 (PMC11073755; doi:10.1093/braincomms/fcae134)

Supplementary Figure 1. Full western blot images for all proteins quantified in the study, including housekeeping proteins.

The ladder is labelled with corresponding molecular weights. Key for first line labels: SCTX = Somatosensory cortex; Blank = Blank lane; P301S = Positive Control; L = Ladder. Key for second line labels: Number is sample number; B = Blank; Ctrl = Positive Control; L = Ladder; Blue = 5.5 month wild-type; Green = 7.5 month wild-type; Orange = 5.5 month rTg4510; Grey = 7.5 month rTg4510. Third line labels are the lane number.

Exclusions due to incomplete or undetectable bands in either the test antibody or GAPDH: Synaptophysin: Samples 35 & 36, PSD95: Samples 35 & 36, GluA1: Sample 36, GluA2: Sample 1, 35, 36, GluA3: Sample 34, GluN1: None.


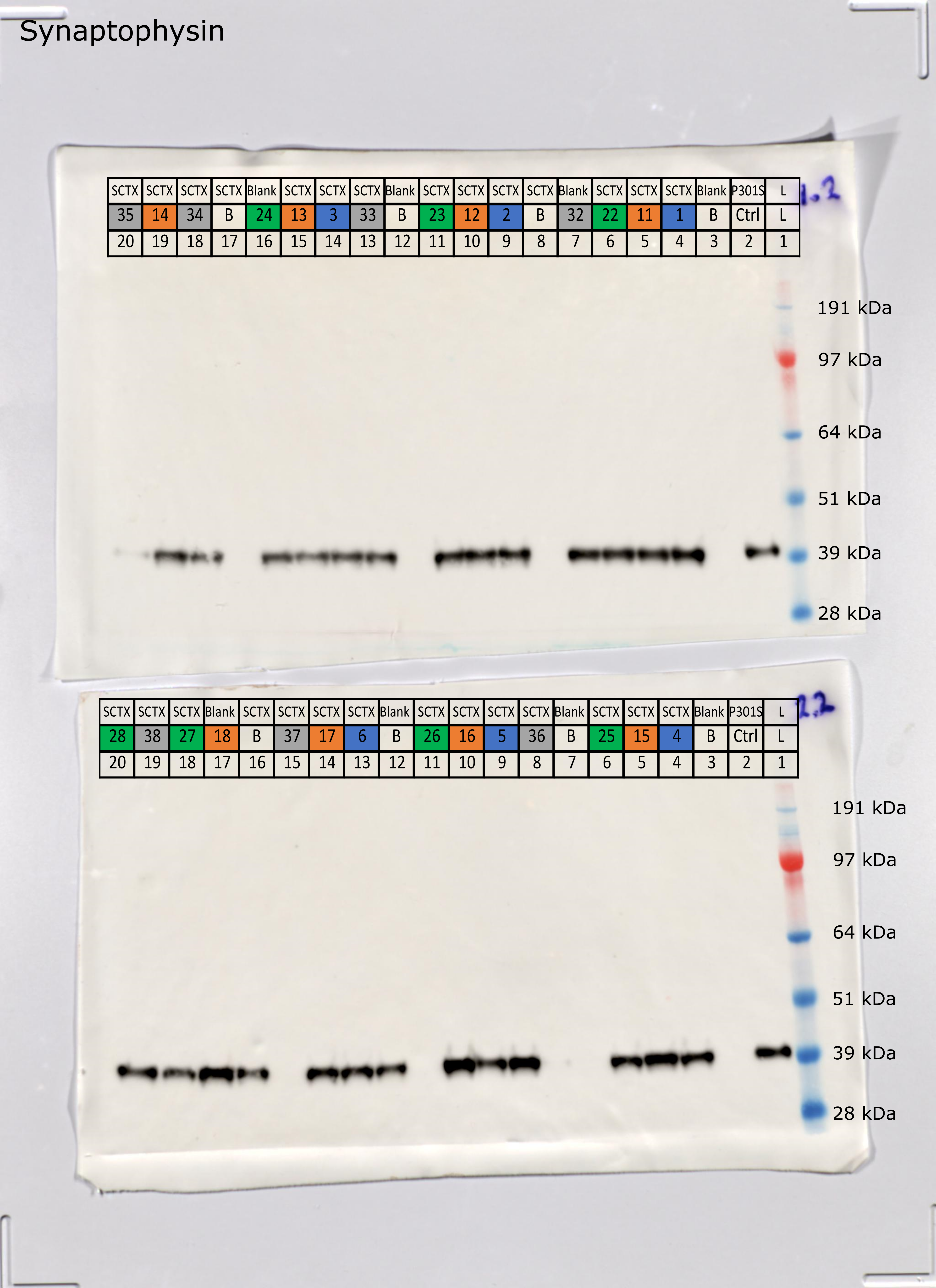

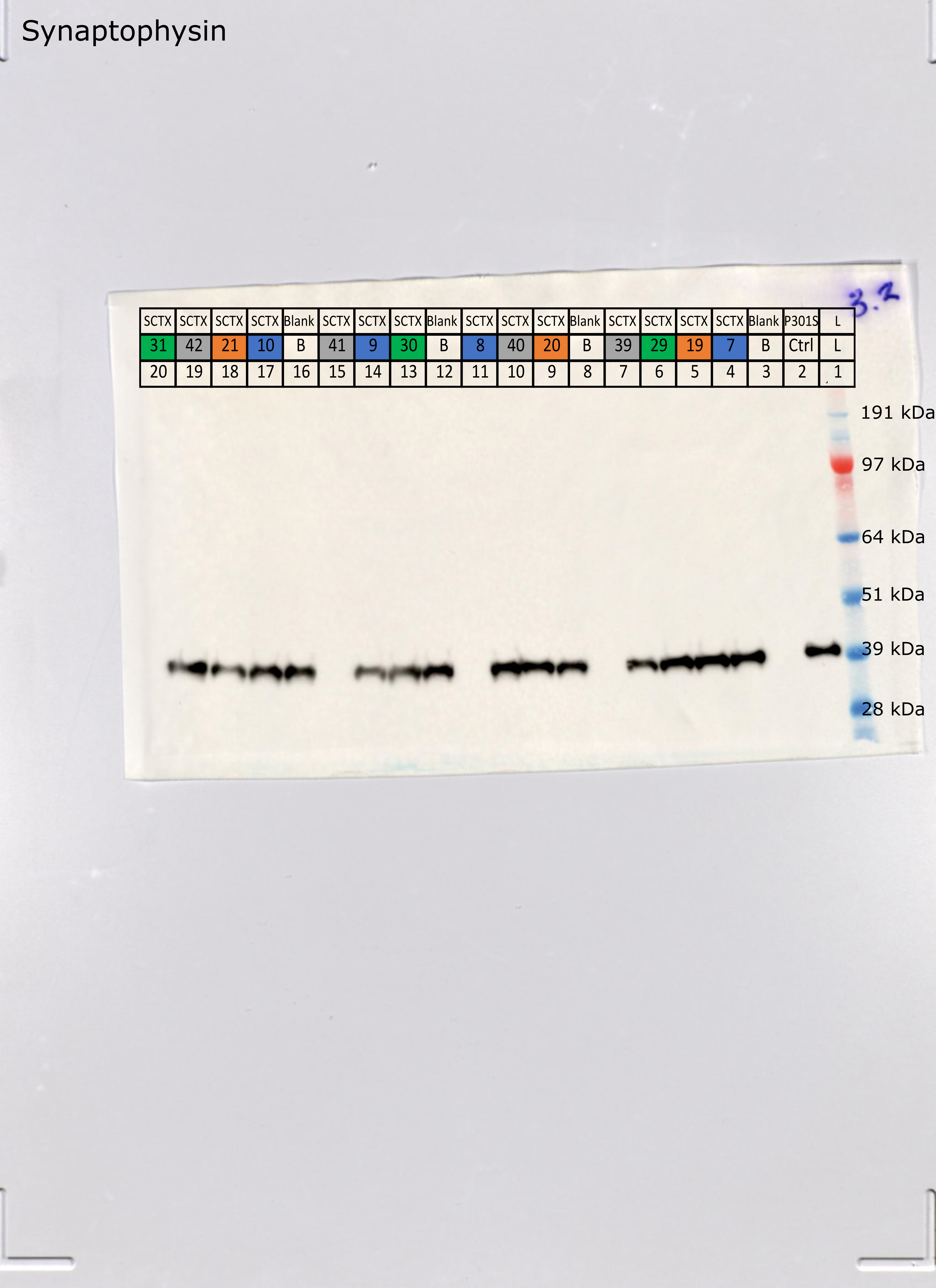

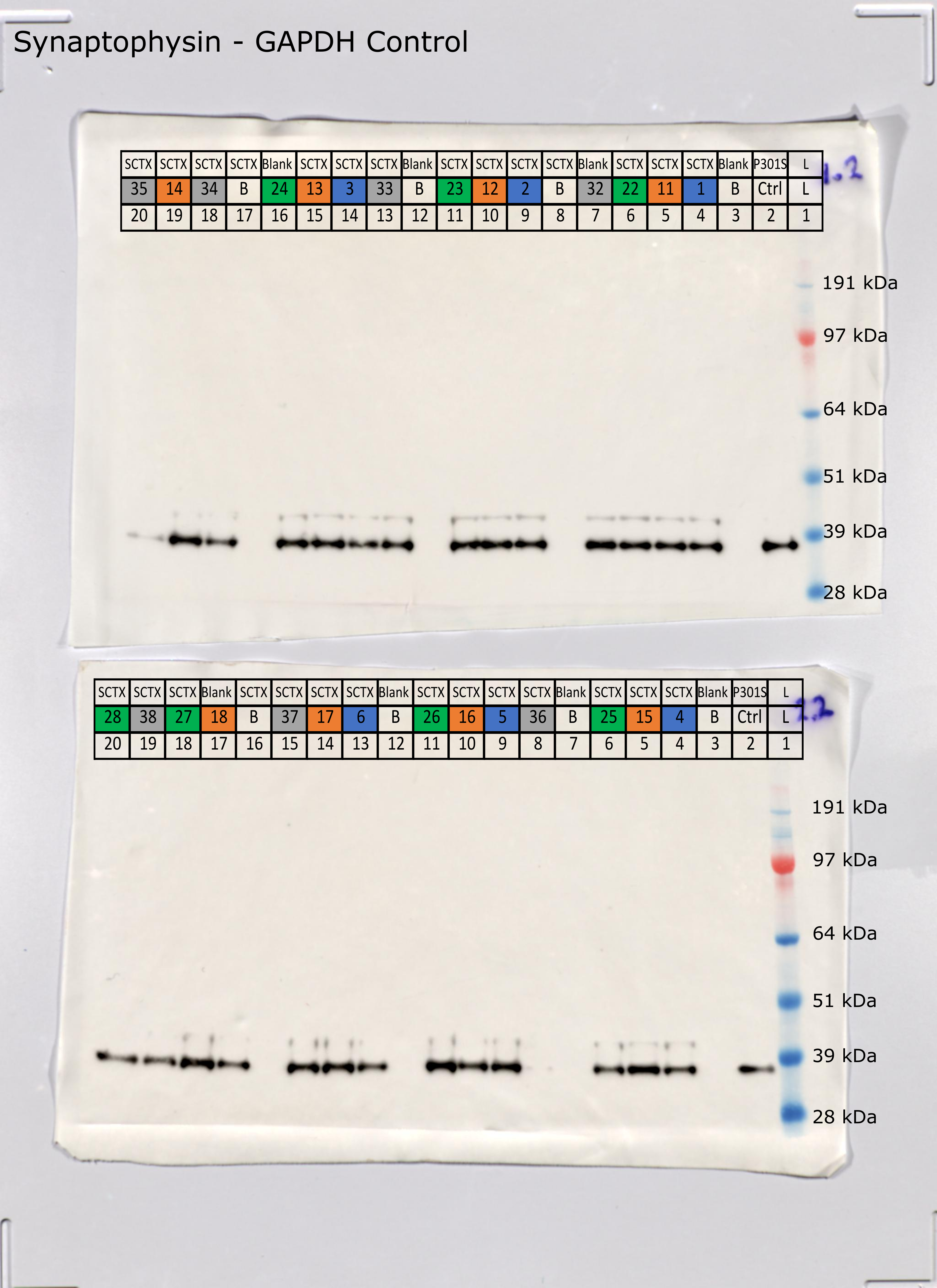

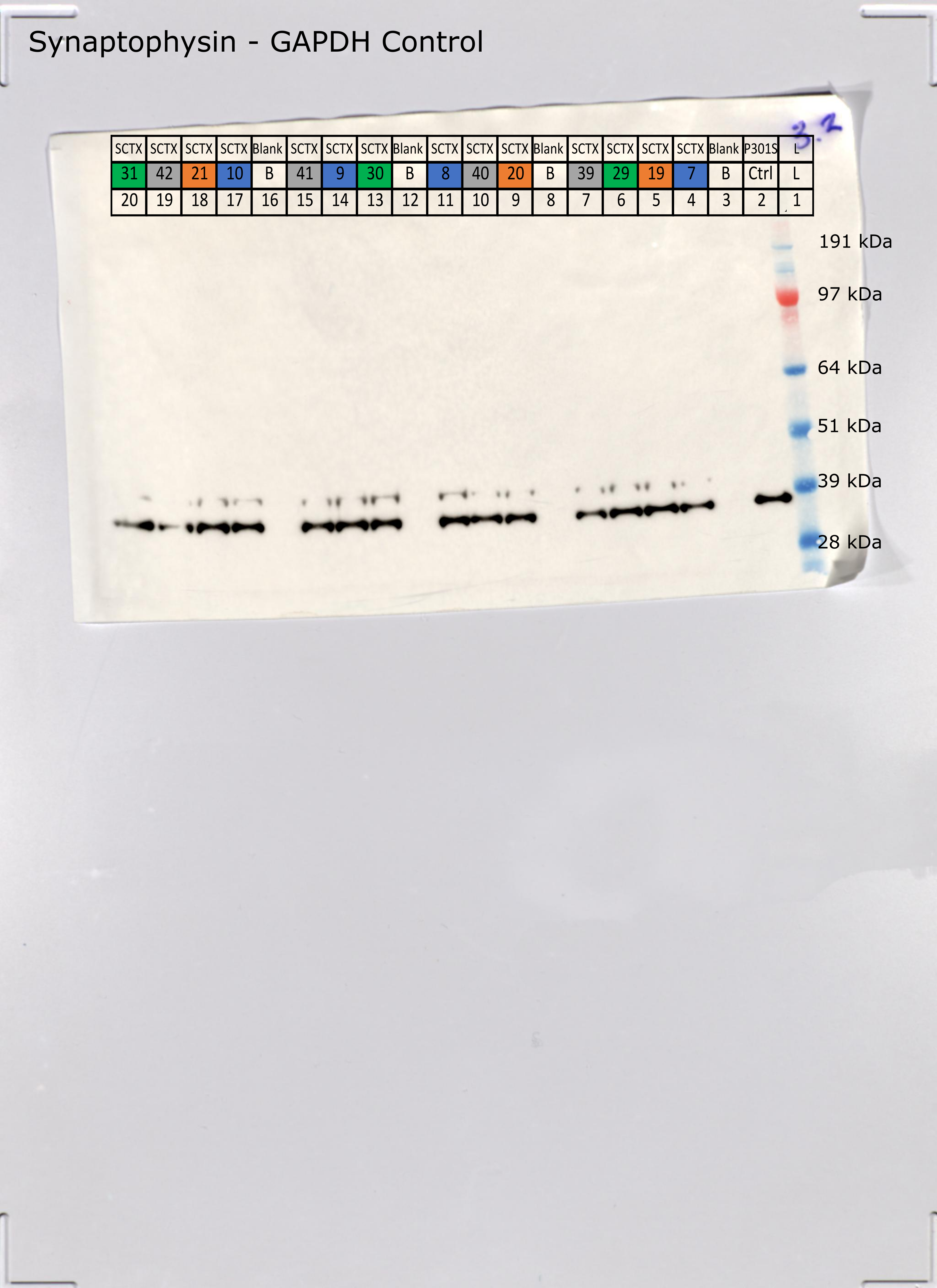

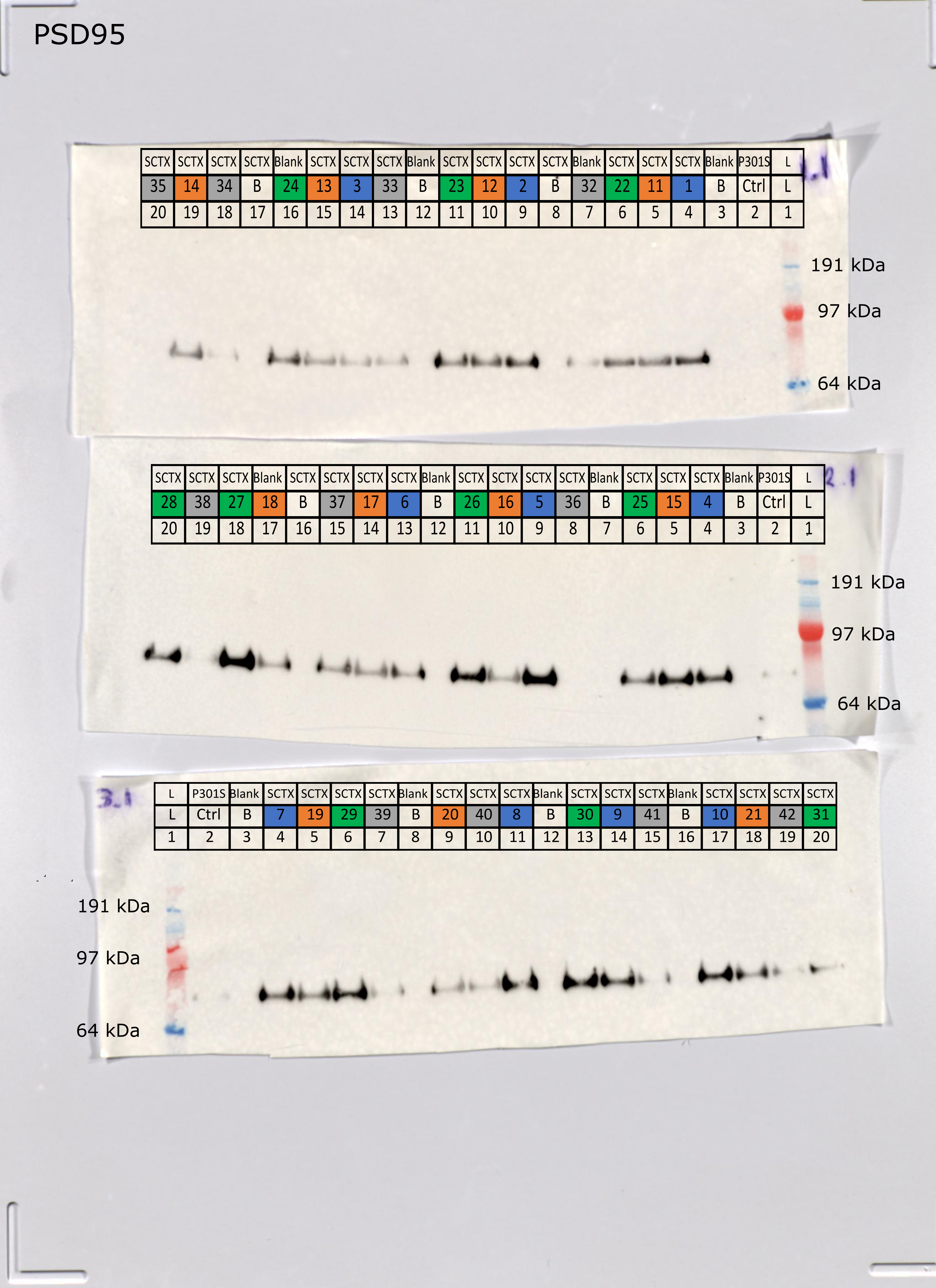

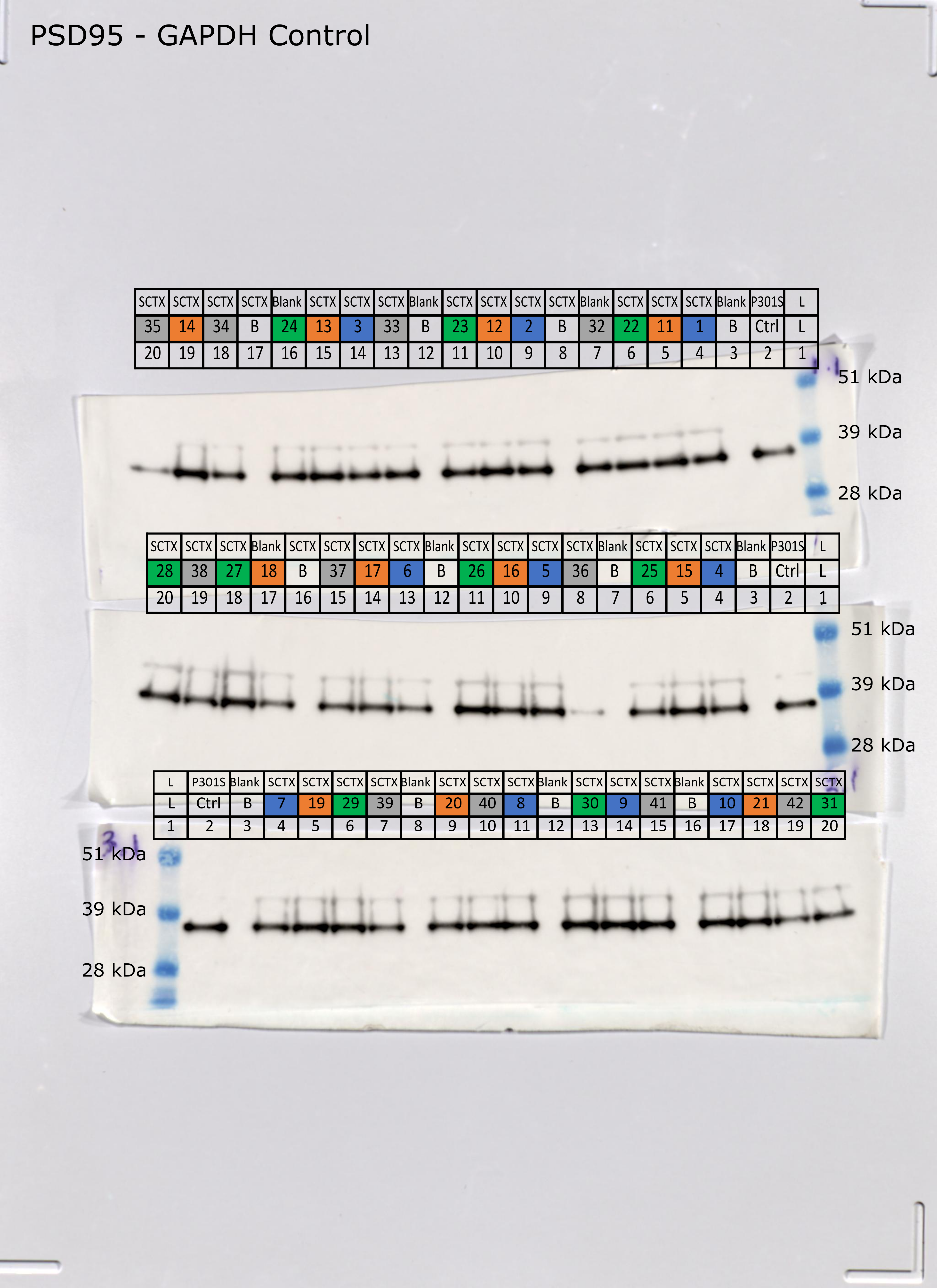

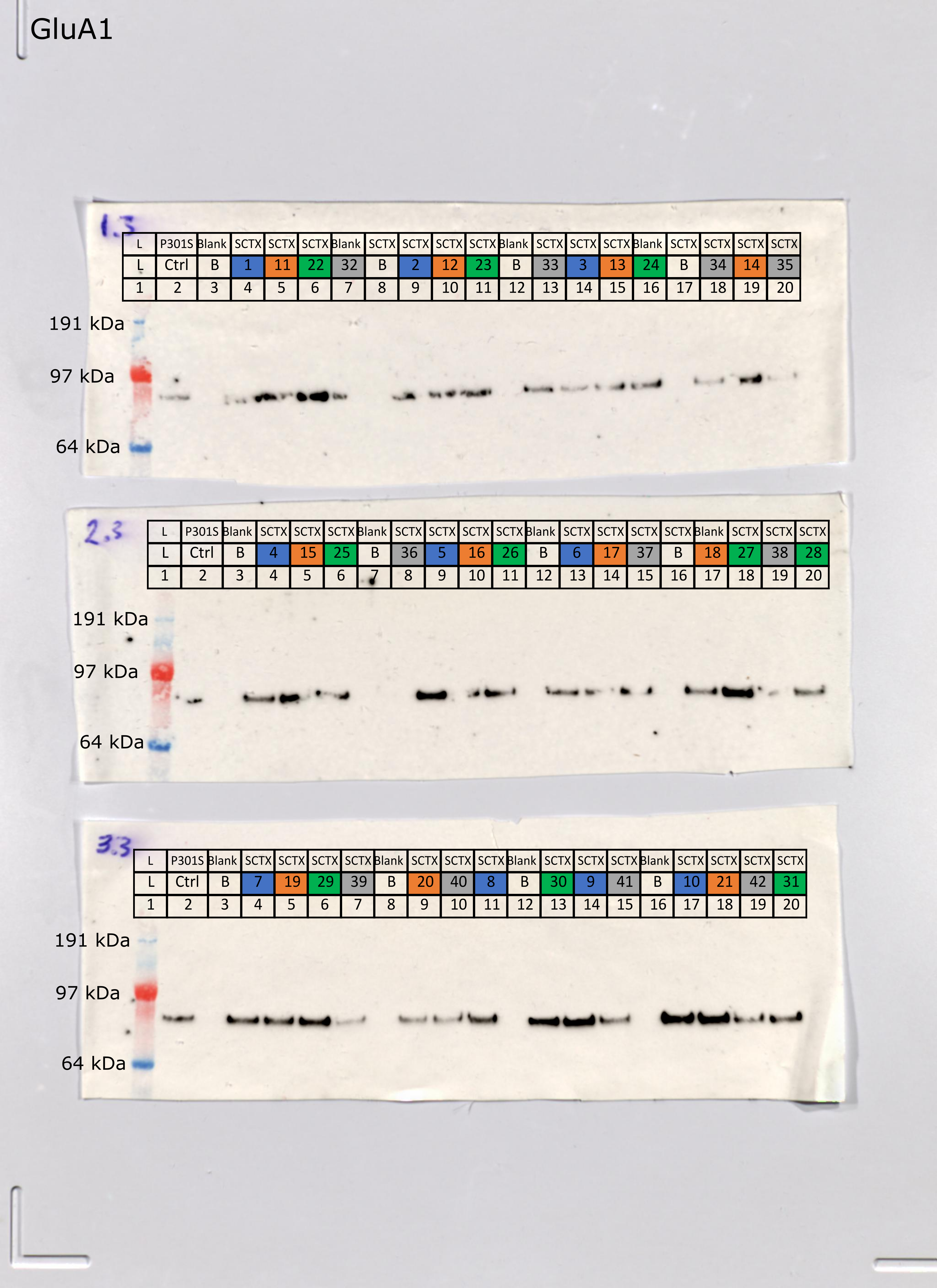

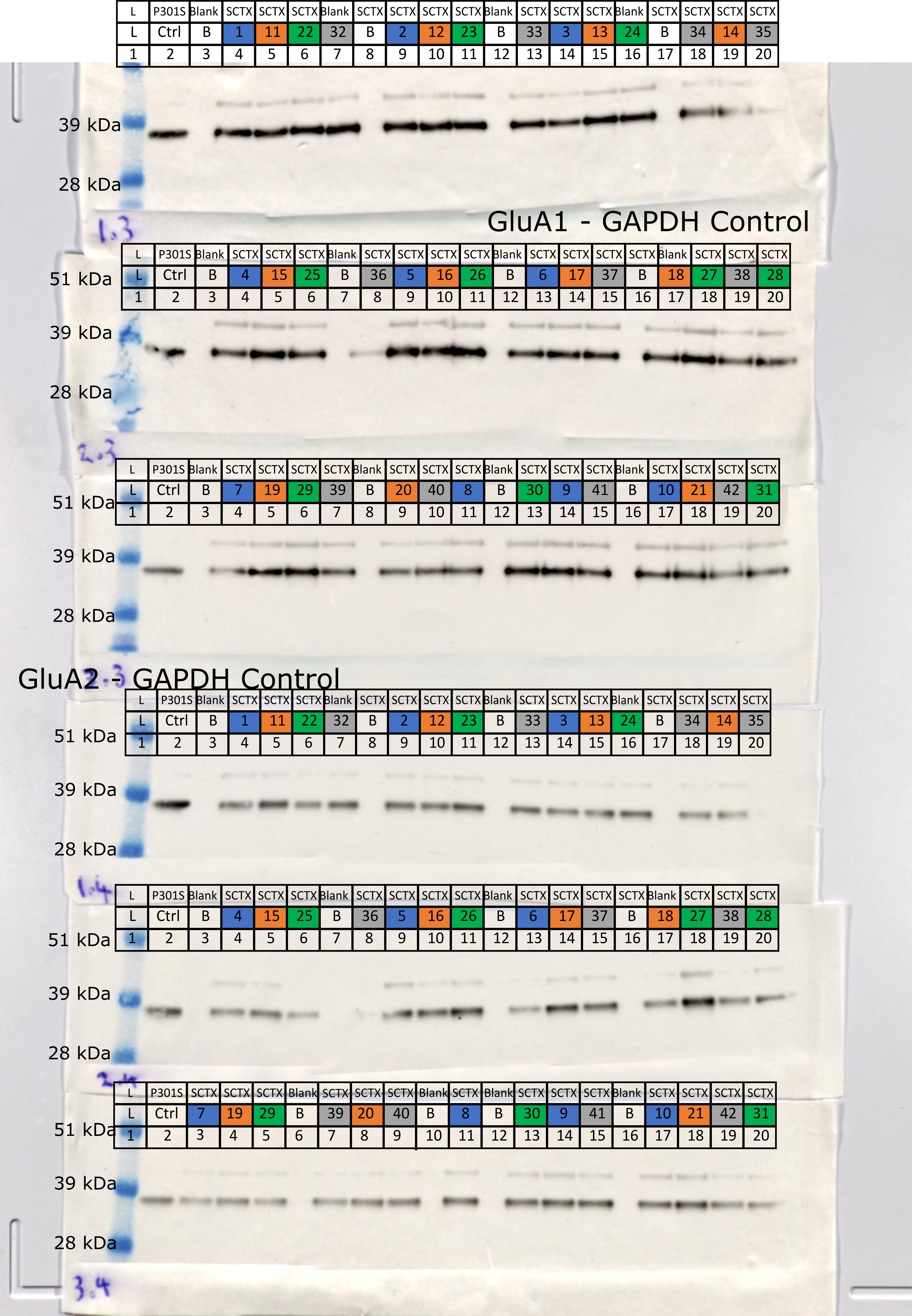

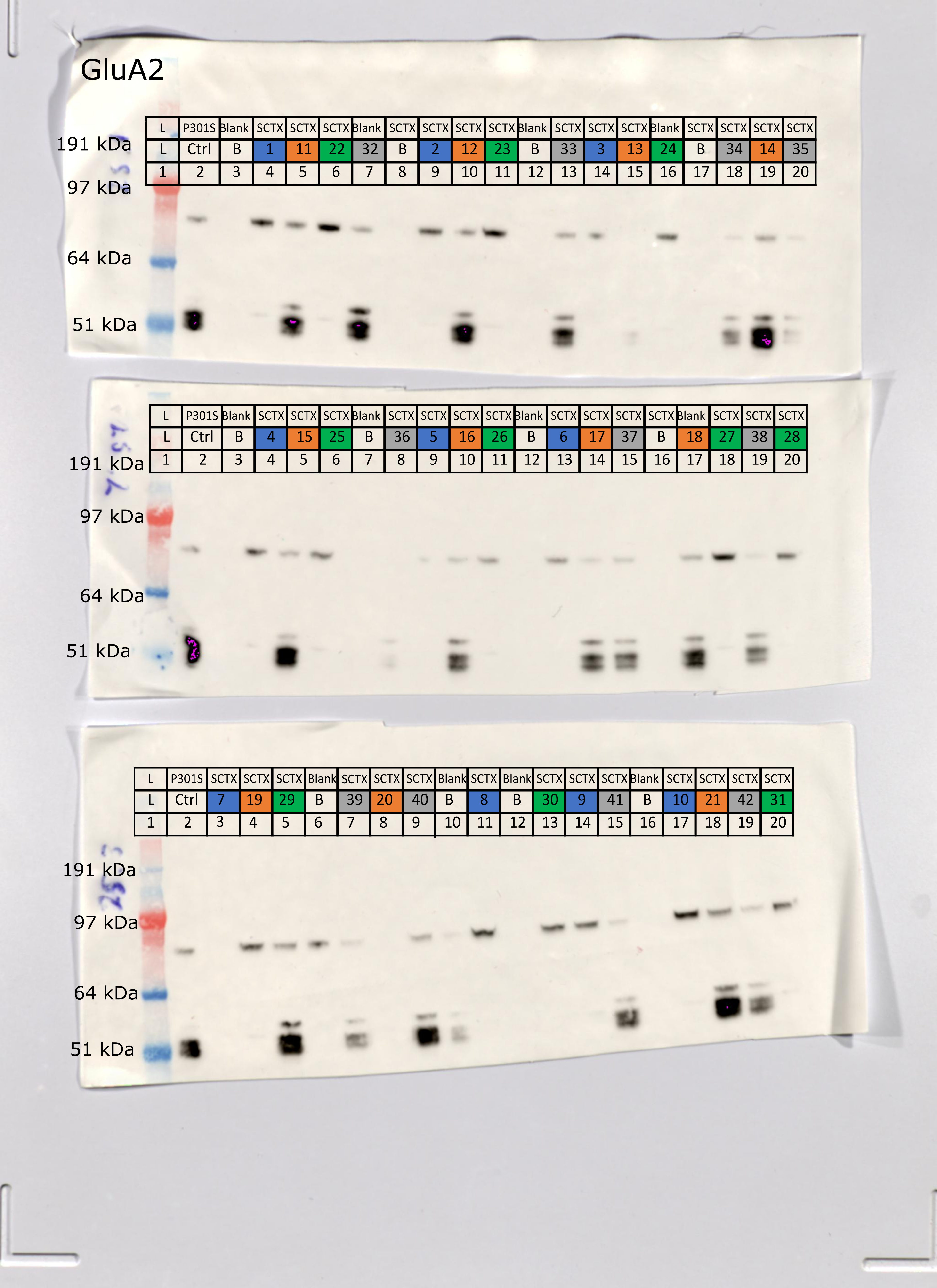

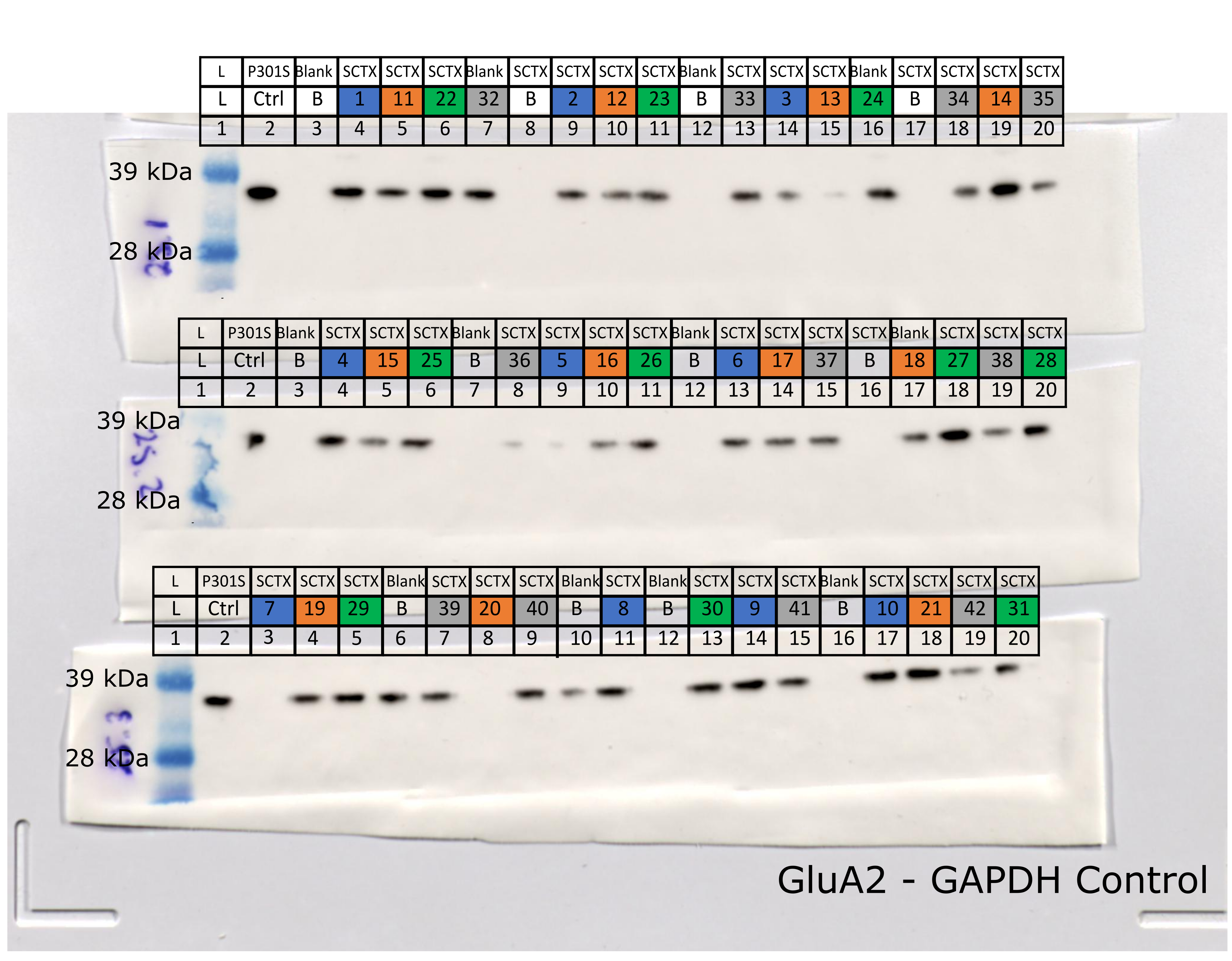

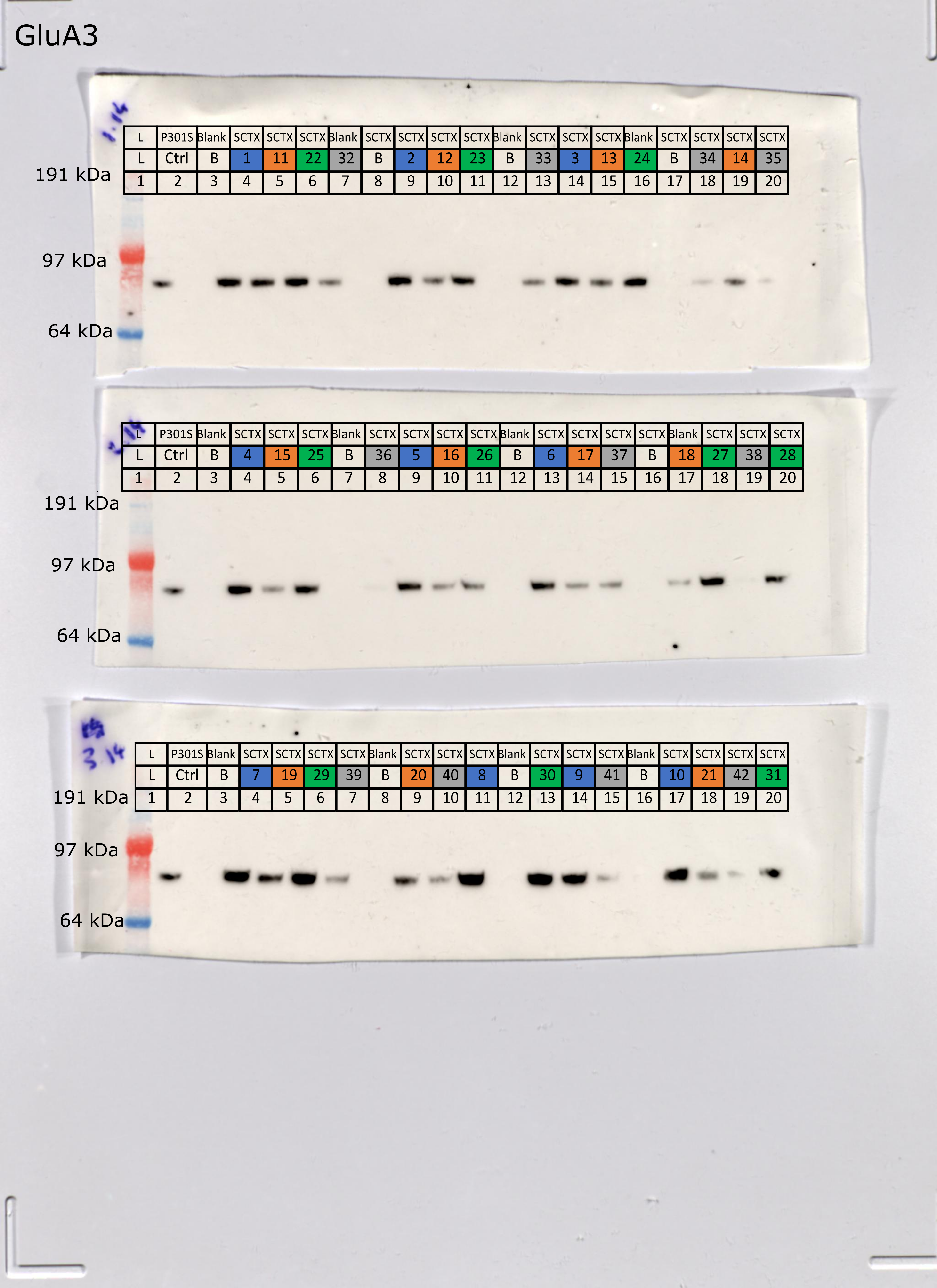

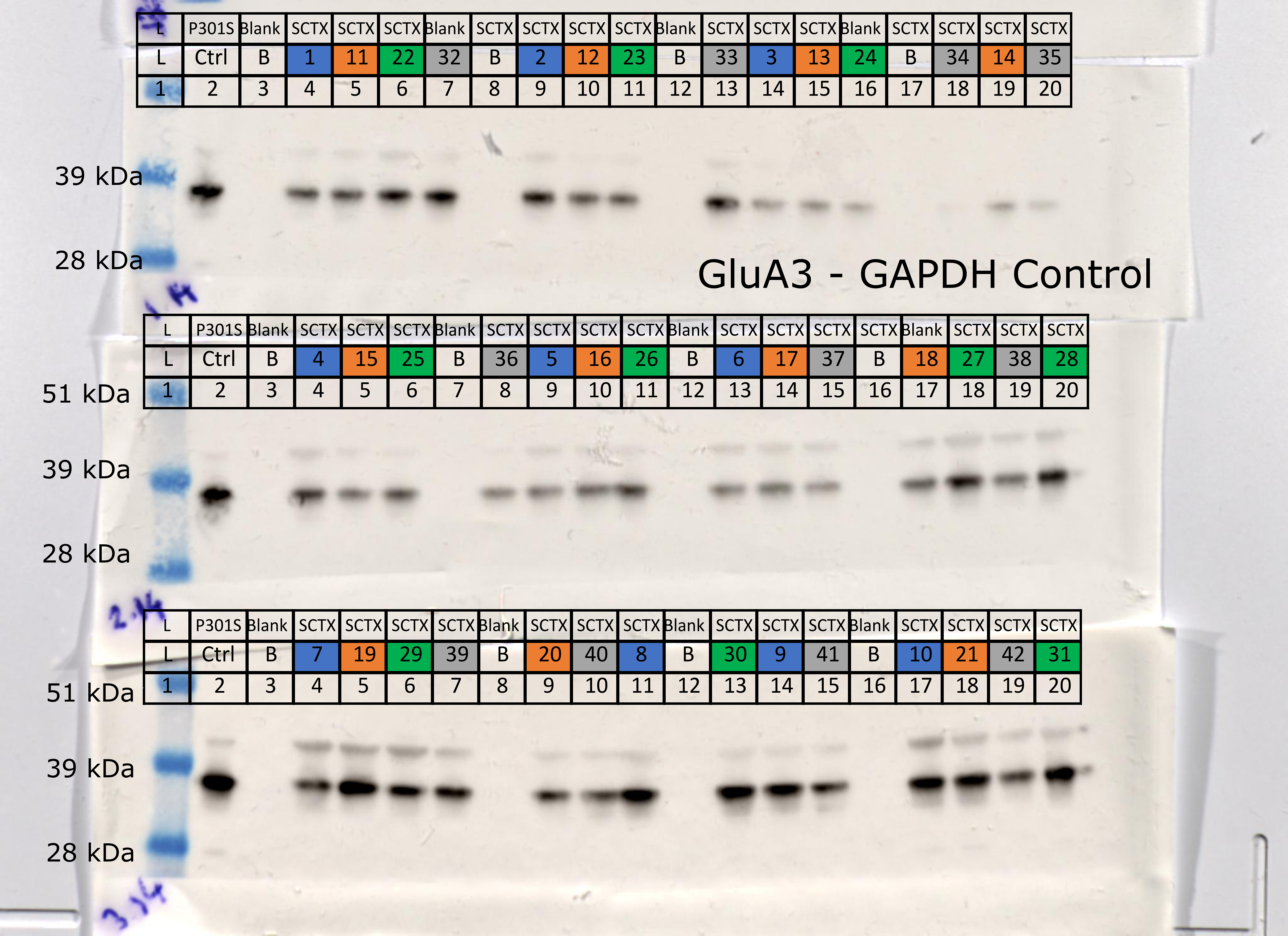

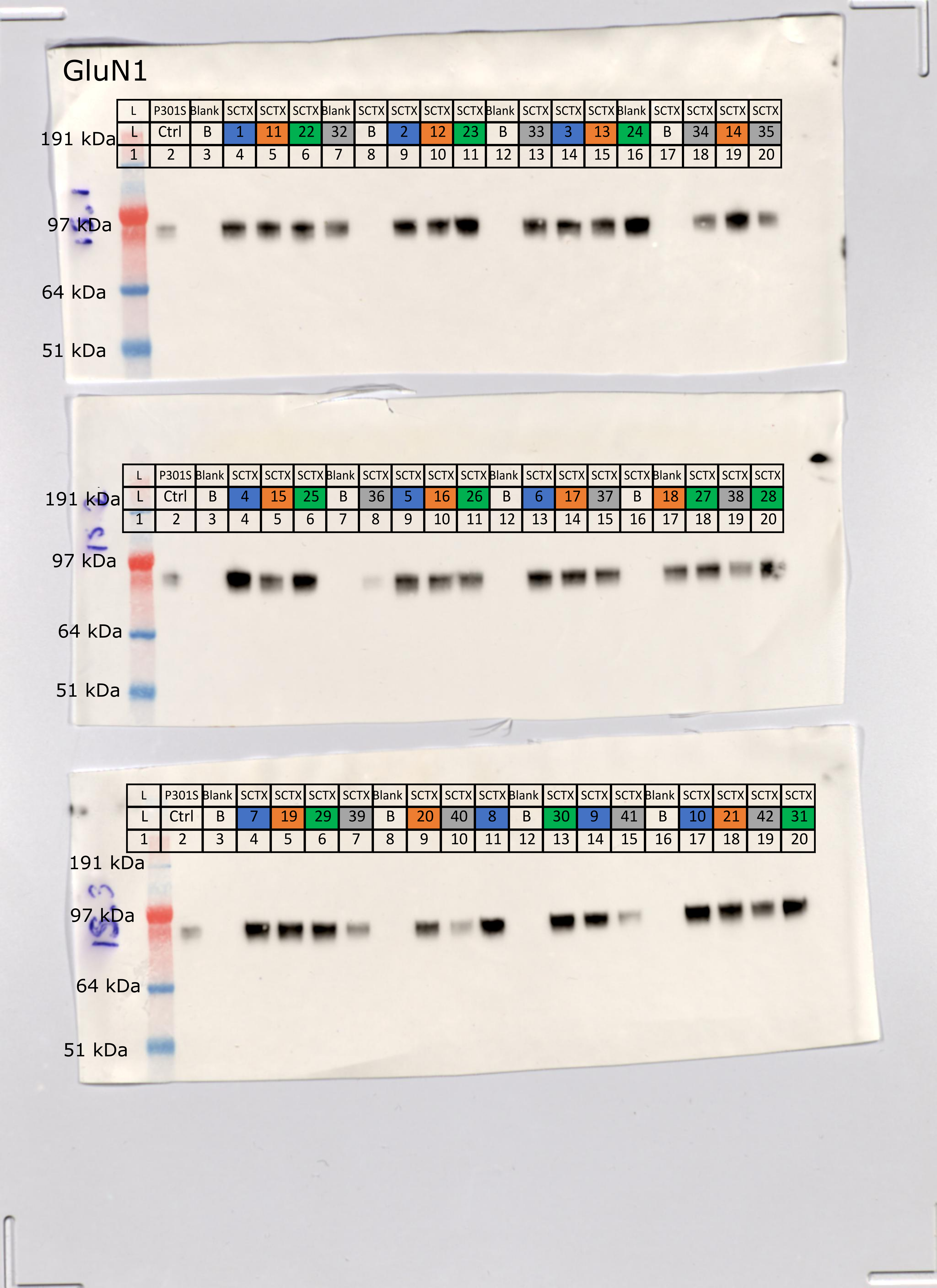

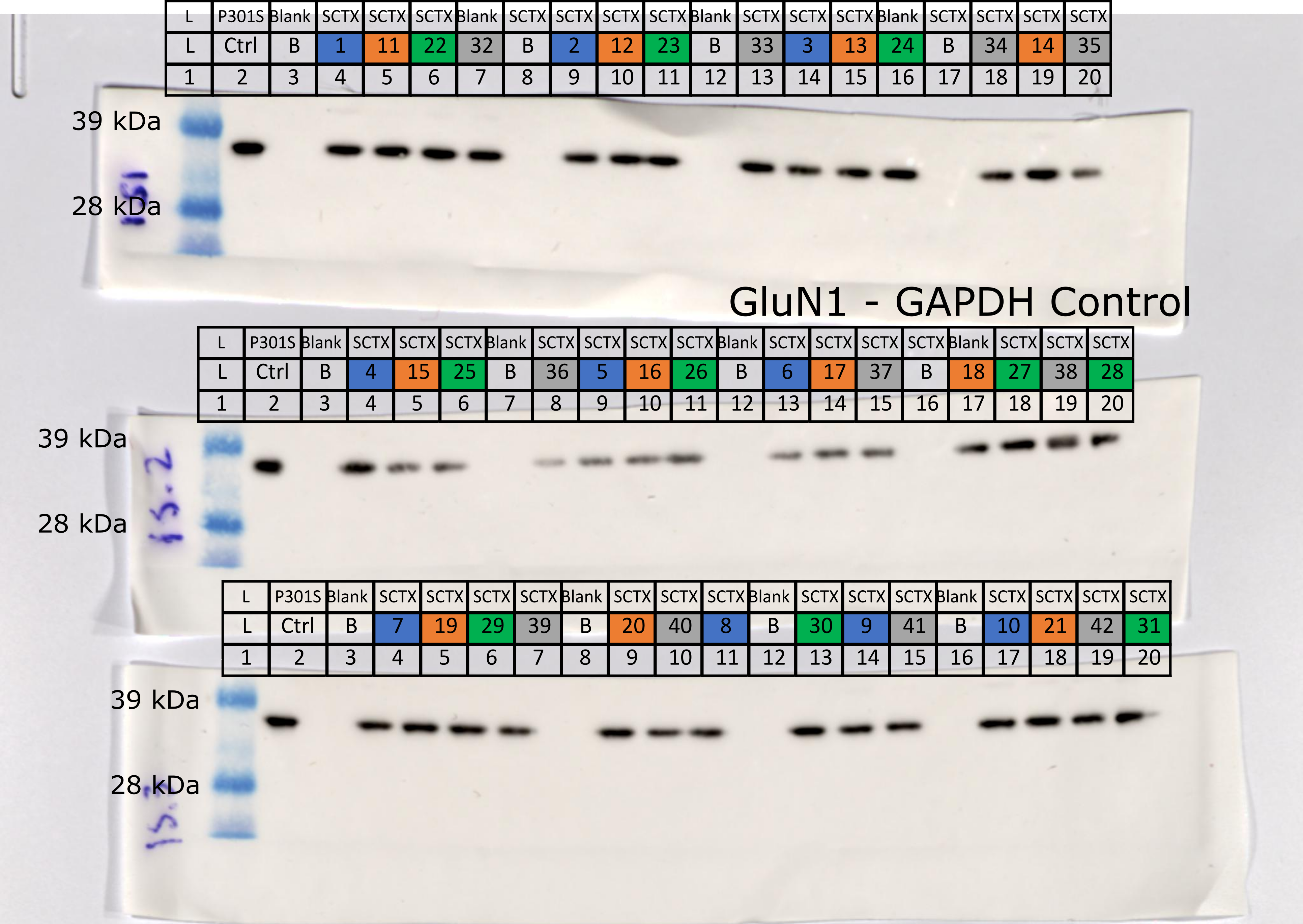

Supplement: fcae134_Supplementary_Data [file fcae134_supplementary_data.zip › Supplementary Figure 1.docx]
