# Supplementary material for: Synaptic alterations associated with disrupted sensory encoding in a mouse model of tauopathy
Source: Brain Commun. 2024 Apr 15;6(3):fcae134. doi: 10.1093/braincomms/fcae134 (PMC11073755; doi:10.1093/braincomms/fcae134)
Supplement: fcae134_Supplementary_Data [file fcae134_supplementary_data.zip › Supplementary_Table 1.docx]

Supplementary Table 1. List of primary and secondary antibodies used in synaptosome experiments

| **Antibody** | **Target** | **Species** | **Dilution used** | **Supplier** | **Catalogue No.** | **Lot No.** |
| --- | --- | --- | --- | --- | --- | --- |
| GAPDH | Housekeeping proteins | Mouse | 1:6000 | Invitrogen | AM4300 | Multiple |
| AT-8 | Tau | Mouse | 1:1000 | Peter Davies | - | - |
| CP27 | Tau | Mouse | 1:1000 | Peter Davies | - | - |
| PSD95 | PSD95 | Mouse | 1:1000 | BD | 610495 | 8128742 |
| Synaptophysin | Synaptic vesicles | Mouse | 1:1000 | Abcam | Ab8049 (SY38) | GR3280108-1 |
| GluR1 (AMPA) [EPR5479] | AMPA receptor, subunit 1 | Rabbit | 1:1000 | Abcam | Ab109450 | GR3241245-1 |
| Anti-GluA2/GluR2 Glutamate Receptor Clone L21/32 | AMPA receptor, subunit 2 | Mouse | 1:1000 | NeuroMab | 75-002 | 472-IJU-17 |
| GluR3 | AMPA receptor, subunit 3 | Mouse | 1:1000 | Invitrogen | 32-0400 | RH240594 |
| NMDAR1 Monoclonal Antibody (54.1) | NMDA receptor, subunit 1 | Mouse | 1:1000 | Invitrogen | 32-0500 | UH286597 |
| Anti-NR2A, M264-10ug | NMDA receptor, subunit 2A | Rabbit | 1:1000 | Sigma | 1002457527 | MKCC8197 |
| NMDAR2B | NMDA receptor, subunit 2B | Mouse | 1:1000 | BD | 610416 | 8159860 |
| GluN2C clone N422/18 | NMDA receptor, subunit 2C | Mouse | 1:1000 | NeuroMab | 75-411 | 455-6JD-37 |
| NR2D | NMDA receptor, subunit 2D | Rabbit | 1:1000 | Abcam | Ab35448 | 851239 |
| HRP linked anti-mouse IgG | Mouse raised immunogens | - | 1:20000 | Cell Signalling | 7076S | Multiple |
| ECL anti-rabbit IgG HRP linked | Rabbit raised immunogens | - | 1:10000 | GE | NA934V | Multiple |
